# Further analysis of tuberculosis in eight high-burden countries based on the Global Burden of Disease Study 2021 data

**DOI:** 10.1186/s40249-024-01247-8

**Published:** 2024-09-30

**Authors:** Hengliang Lv, Longhao Wang, Xueli Zhang, Caixia Dang, Feng Liu, Xin Zhang, Junzhu Bai, Shumeng You, Hui Chen, Wenyi Zhang, Yuanyong Xu

**Affiliations:** 1 Department of Epidemiology, School of Public Health, China Medical University, Shenyang, China; 2 Chinese People’s Liberation Army Center for Disease Control and Prevention, Beijing, China; 3 Department of Health Statistics, Faculty of Military Preventive Medicine, Army Medical University, Chongqing, China; 4 Changchun University of Chinese Medicine, Changchun, China

**Keywords:** Tuberculosis, High-Burden countries, Age-period-cohort model, Bayesian age-period-cohort, Prediction

## Abstract

**Backgrounds:**

Most significant findings from the Global Tuberculosis (TB) Report 2023 indicate that India, Indonesia, China, the Philippines, Pakistan, Nigeria, Bangladesh, and the Democratic Republic of the Congo (DRC) collectively contribute to approximately two-thirds of global TB cases. This study aims to provide crucial data-driven insights and references to improve TB control measures through a comprehensive analysis of these eight high-burden countries.

**Methods:**

The eight high-burden TB countries analyzed in this study include India, Indonesia, China, the Philippines, Pakistan, Nigeria, Bangladesh, and the DRC. Age-standardized incidence rates (ASIR) of TB were derived from the Global Burden of Diseases Study 2021 data. Temporal trends were analyzed using Joinpoint regression. An age-period-cohort model was applied to examine the risk ratios (RR) of TB across diverse age groups, periods, and birth cohorts. A Bayesian age-period-cohort framework was employed to predict the ASIR of TB by 2030.

**Results:**

The study found that the Philippines (average annual percentage change = 3.1%, *P* < 0.001) exhibited an upward trend from 1990 to 2021. In India, the Philippines, Pakistan, and Bangladesh, the RR of TB incidence exceeded 1 after individuals reached 25 years old. Notably, the RR has shown a consistent upward trend since 2001, peaking during the period of 2017–2021 with an estimated RR of 1.5 (*P* < 0.001) in the Philippines. Similarly, the highest RR was observed during the period of 2017–2021 reaching 1.1 (*P* < 0.001) in the DRC. In the Philippines, the markedly increasing RR values for TB have been observed among individuals born after 1997–2001. Projections suggest that the ASIR of TB is expected to follow a continued upward trajectory, with an estimated rate of 392.9 per 100,000 by 2030 in the Philippines; India and Indonesia are projected to achieve less than 20.0% of the target set by the World Health Organization (WHO).

**Conclusions:**

Among the eight high-burden countries, the Philippines, India and Indonesia are diverging from the goals set by the WHO, and the risk of TB in the Philippines and the DRC shows a trend toward affecting younger populations, which suggests that the management strategies for TB patients need to be further strengthened.

**Graphical Abstract:**

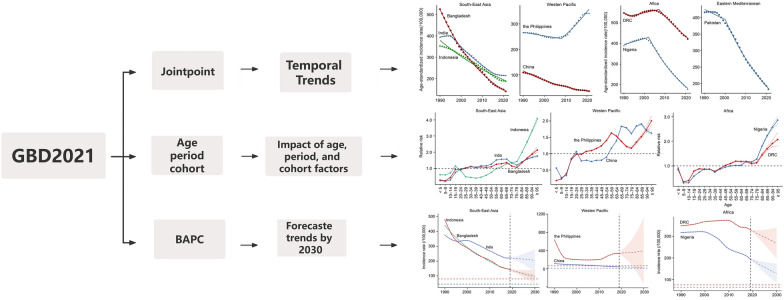

**Supplementary Information:**

The online version contains supplementary material available at 10.1186/s40249-024-01247-8.

## Background

Tuberculosis (TB) is an ancient disease that has been affected humans for thousands of years [1]. Despite being preventable and generally curable, TB remained the world’s second-leading cause of death from a single infectious agent in 2022, after coronavirus disease 2019 (COVID-19), and caused nearly twice as many deaths as human immunodeficiency virus(HIV)/acquired immune deficiency syndrome(AIDS) [2]. It was estimated that about a quarter of the world’s population was infected with *Mycobacterium tuberculosis* [3], with approximately 10.6 million new cases of TB and 1.3 million deaths attributed to TB estimated in 2022, placing a severe burden on global health [4].

It is crucial to recognize that preventing the further spread of TB is essential for alleviating the suffering caused by this disease. However, the severity of TB epidemics varies significantly across countries, with effective control of TB in high-burden countries is crucial. According to the Global TB report 2023, the eight countries mentioned accounted for two-thirds of the global TB cases in 2022: India (27.0%), Indonesia (10.0%), China (7.1%), the Philippines (7.0%), Pakistan (5.7%), Nigeria (4.5%), Bangladesh (3.6%), and the Democratic Republic of the Congo (DRC) (3.0%), which were located in the WHO Southeast Asian, African, and Western Pacific regions. It is worth noting that most of these countries are low- or middle-income countries with large population bases, contributing to the severe imbalance in health resource allocation [5]. Therefore, targeted allocation of health resources to high-risk TB groups in low-development regions is particularly important [6]. Previous research has found that demographic differences may affect susceptibility to TB biologically and socially, with older individuals being more susceptible to TB than younger ones [7, 8], however, there has been a shift in the age structure of TB cases in recent years, and differences in nutritional status and environmental factors across birth cohorts may also lead to changes in the risk of developing TB in subsequent generations [9]. Despite this, there is currently a lack of information on the relationship between TB incidence and age, period, or birth cohort in eight high-burden countries. Furthermore, it has been noted that many regions and countries affiliated with the World Health Organization (WHO) did not meet the initial objectives set for 2020. The secondary goal remains out of reach in many regions globally. The challenges posed by the COVID-19 pandemic and demographic shifts have further complicated the attainment of these critical milestones. Therefore, quantifying the projected TB incidence in the eight high-burden countries and evaluating progress towards the second target could provide crucial insights for adapting future TB control strategies.

Based on this, this study employed the age-period-cohort model to further analyze the characteristics of TB patients in these countries, and utilized the Bayesian age-period-cohort (BAPC) model to predict the TB incidence and quantitatively assess the gap to the target set by the WHO for 2030, using data from the Global Burden of Diseases, Injuries, and Risk Factors Study (GBD) 2021. The aim was to provide data support and reference for achieving the goal of “ending TB” as soon as possible.

## Methods

### Overview

The eight high-burden TB countries analyzed in this study include India, Indonesia, China, the Philippines, Pakistan, Nigeria, Bangladesh, and the DRC. The data used in this study, including the incidence of TB in eight high-burden countries and total population data by age group, comes from the GBD 2021, compiled by the Institute for Health Metrics and Evaluation. The GBD 2021 offers comprehensive and current estimates of health burdens, categorized by age, gender, and country, covering 371 diseases and injuries across 204 countries and territories. The methods used in GBD 2021 closely followed those used in GBD 2019 [10], and the GBD analytical framework for TB, along with the methods for TB data collection, have been previously detailed in other literature [5]. The data used in this study can be retrieved from: https://vizhub.healthdata.org/gbd-results/. Additionally, to assess the situation in high-burden countries across the WHO regions, subsequent analysis and data visualization were conducted on a region-by-region basis [4].

### TB morbidity

The Disease-model-Bayesian meta-regression (DisMod-MR) was used in our study to simultaneously model age-sex-specific TB incidence, prevalence, and cause-specific mortality. This is a modeling tool that leverages all available morbidity and mortality data, the epidemiological relationships between disease parameters, and spatial relationships to provide internally consistent estimates of the disease burden [10]. The brief methods for estimating TB morbidity are as follows: First, TB cases in the GBD 2021 study were identified through a thorough review of population-based TB prevalence surveys in the literature. A Bayesian meta-regression tool [11] was employed to adjust prevalence surveys that relied on smear-positive TB as the case definition, instead of bacteriologically confirmed TB, and then recalibrated surveys that used symptoms only as the screening method over both symptoms and chest X-ray. Next, the study maximized the data informing DisMod-MR by predicting age-sex-specific incidence for countries with low-quality ratings for causes of death data. For these countries the incidence was estimated using a mortality-to-incidence (MI) ratio approach, which utilized countries with high-quality cause of death data as input for a Bayesian meta-regression analysis, where the primary covariate was the Health Assessment Questionnaire index [12]. The location-specific predicted MI ratios were then linked with TB death estimates to obtain the estimated age-sex-specific incidence. Finally, these data, along with age-sex-specific case notifications for locations with high-quality causes of death data, population-based tuberculin surveys, and estimates of TB excess mortality rate, remission, and cause-specific mortality rate, were used to generate all-form TB morbidity estimates via DisMod-MR, ensuring internal consistency. The study disaggregated TB incidence by HIV status by applying the fraction of all-form TB cases that were HIV and TB co-infected to the all-form TB cases estimated from DisMod-MR. More detailed TB incidence estimates are presented in other articles [13].

### Temporal analysis

The Joinpoint regression analysis was used to analyze the temporal trends of the TB incidence rate from 1992 to 2021 in eight high-burden TB countries. This method identifies significant joinpoints, which indicate substantial changes in the trend. The annual percentage change (APC) and average annual percentage change (AAPC) were used to analyze incidence trends. A positive APC/AAPC estimate with the lower boundary of its 95% confidence interval (*CI)* greater than zero, indicated an upward trend. Conversely, if the APC/AAPC estimate and the upper boundary of its 95% *CI* were both below zero, a downward trend was observed. If neither condition was met, the trend was considered stable. For a more detailed explanation of Joinpoint regression, please refer to other articles [14] or visit: https://surveillance.cancer.gov/joinpoint/.

### Age-period-cohort model

This study grouped the number of TB cases and the population counts for each five-year age group, then merged them. Birth cohorts were calculated using the formula “cohort = period—age”, and the data was formatted accordingly. The age-period-cohort model for eight high-burden TB countries was constructed using the “apc” package in Stata software (version 17.0; StataCorp LLC, Texas, USA). The analysis examined the impact of age, period, and cohort factors on TB incidence. To address collinearity issues in the age-period-cohort model, the intrinsic estimator (IE) method proposed by Yang et al. [15] was employed. A log-linear model was used to estimate the coefficients, as follows: *ln*(*y*_*ijk*_*/n*_*ijk*_) = *μ* + *α*_*i*_ + *β*_*j*_ + *γ*_*k.*_ In the model, *y*_*ijk*_*/n*_*ijk*_ represents the incidence rate and *μ* represents the intercept. The indices *i*, *j*, and *k* represent age groups, period groups, and birth cohort groups, respectively. *α*_*i*_, *β*_*j*_, and *γ*_*k*_ represent the regression coefficients for each age, period, and birth cohort group. The estimated coefficients were used to calculate the relative risk (RR) of TB incidence by substituting them into the exponential function, i.e., RR = exp (coefficient). This model has also been widely used in other studies [7, 16].

### BAPC model

The study employed the BAPC model to analyze the effects of age, period and cohort on the age-standardized incidence rate (ASIR) of TB and forecast trends by 2030. According to Bayes’ theorem, posterior information is obtained by synthesizing prior information and the sample data to infer unknown parameters. The BAPC model is based on second-order random walk (RW2) model, which smooths the prior age, period, and cohort effects to derive posteriori incidence [17]. RW2 assumes that second-order differences in temporal effects followed independent zero-mean normal distributions [18]. Additionally, Markov Chain Monte Carlo (MCMC) iteration was used to stabilize the posterior probability distribution, ensuring better coverage and accuracy for the BAPC model [19]. To optimize computational efficiency and manage high-dimensional data, Integrated Nested Laplace Approximation was employed within the Bayesian framework as an alternative to traditional MCMC methods [20]. Notably, to eliminate the impact of the COVID-19 pandemic on TB [21], this study forecasted TB incidence starting from 2020.

### Statistical analysis

For temporal trend analysis, Joinpoint regression software (version 4.9.1.0; Statistical Methodology and Applications Branch, Surveillance Research Program, National Cancer Institute, Bethesda, MD, USA) was used. The APC and BAPC model were executed using R software (version 4.2.1; R Foundation for Statistical Computing, Vienna, Austria). All visualization tasks were performed using R software (version 4.2.1). A *P*-value less than 0.05 was considered statistically significant.

## Results

### Temporal analysis of TB incidence for eight high-burden countries

**Fig. 1:**
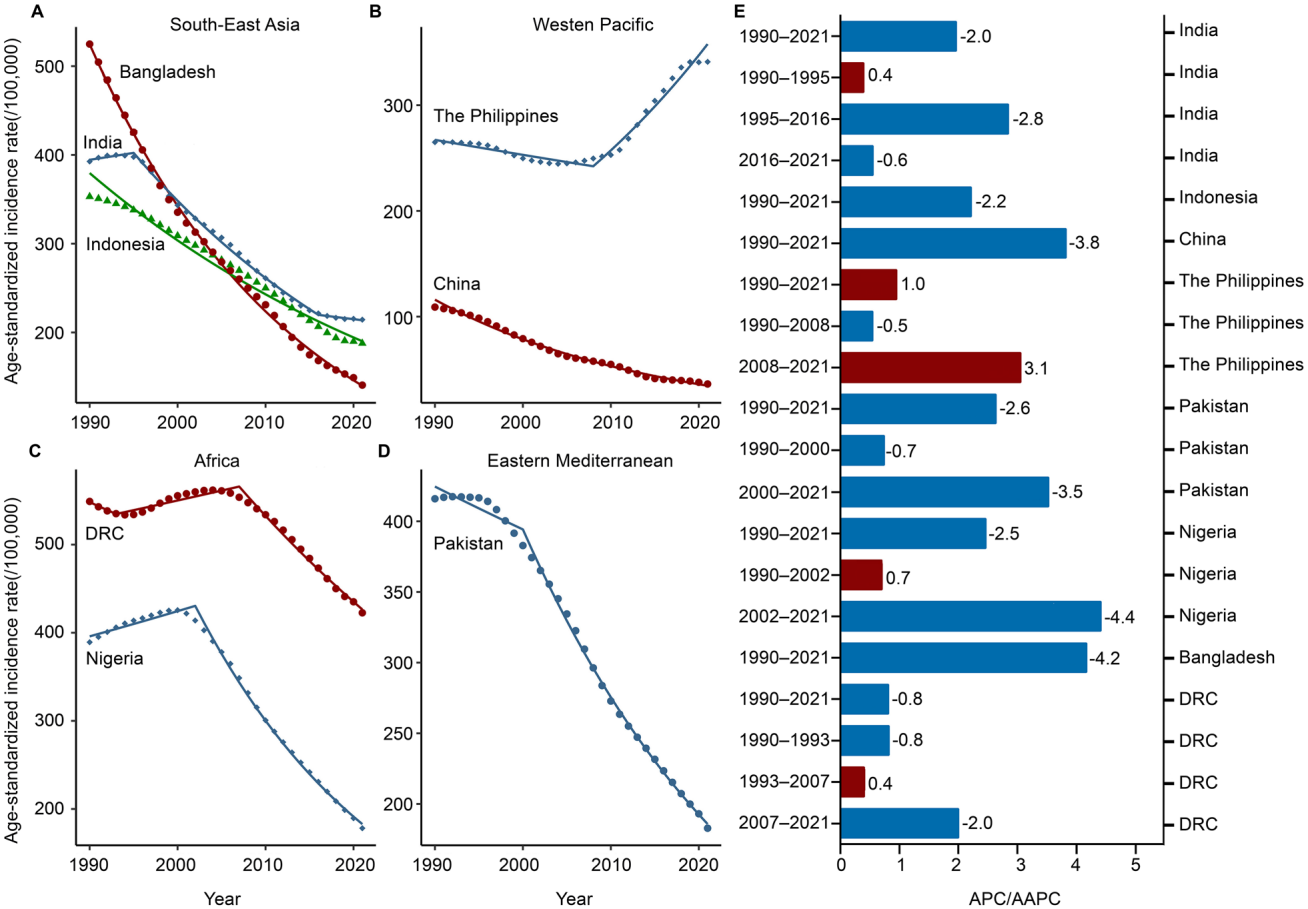
Temporal analysis of age-standardized tuberculosis incidence rates in eight high-burden countries from 1990 to 2021. **A** High-burden tuberculosis countries in the Southeast Asia Region according to the World Health Organization. **B** High-burden tuberculosis countries in the Western Pacific Region according to the World Health Organization. **C** High-burden tuberculosis countries in the Africa Region according to the World Health Organization. **D** High-burden tuberculosis countries in the Eastern Mediterranean Region according to the World Health Organization. **E** APC and AAPC for tuberculosis temporal trends in eight high-burden countries. *APC* annual percentage change; *AAPC* average annual percentage change; *DRC* the Democratic Republic of the Congo

The estimated number of cases in India was 2,955,264 cases (ASIR: 214.39/100,000), China had 617,725 cases (ASIR: 36.28/100,000), and Indonesia had 488,085 cases (ASIR: 187.76/100,000). These three countries accounted for the highest number of cases globally in 2021 (Table 1). Figure 1 illustrates a sustained downward trend in TB ASIR across most countries from 1990 to 2021. For instance, Bangladesh experienced a decrease from 524.87/100,000 to 140.67/100,000 (AAPC = − 4.2%, *P* < 0.001), China’s rate declined from 109.01/100,000 to 36.28/100,000 (AAPC = − 3.8%, *P* < 0.001), and Pakistan’s ASIR dropped from 415.94/100,000 to 182.83/100,000 (AAPC = − 2.6%,* P* < 0.001). However, it is worth noting that the Philippines (AAPC = 3.1%, *P* < 0.001) experienced an upward trend during the study period. Over the years, an upward trend was observed in two countries: Nigeria from 1990 to 2002 (APC = 0.7%, *P* < 0.001) and the DRC from 1993 to 2007 (APC = 0.4%, *P* < 0.001), both located in the WHO African region. Additionally, the Philippines showed the most pronounced growth trend from 2008 to 2021 (APC = 3.1%, *P* < 0.001) (Fig. 1A–E & Additional file 1: Table S1).

**Table 1:** Tuberculosis incidence in eight high-burden tuberculosis countries in 1990, 2019, and 2021

Country	1990		2019		2021	
	Number (95% UI)	ASIR (95% UI) per 100,000	Number (95% UI)	ASIR (95% UI) per 100,000	Number (95% UI)	ASIR (95% UI) per 100,000
India	265,6904 (217,3904–326,2441)	392.62 (323.90–477.11)	285,2870 (249,0728–326,5367)	215.14 (188.70–244.67)	295,5264 (259,9262–338,3727)	214.39 (188.98–244.86)
Indonesia	53,5619 (47,6392–60,4907)	352.02 (318.72–392.04)	47,2895 (42,5388–52,2745)	190.60 (171.05–208.56)	48,8085 (43,7711–54,0514)	187.76 (168.64–205.63)
China	116,7807 (100,4441–135,9621)	109.01 (94.81–124.61)	64,6239 (57,6192–71,8146)	39.00 (35.10–43.21)	61,7725 (54,9548–68,8348)	36.28 (32.63–40.47)
The Philippines	11,7228 (10,5248–13,2405)	265.00 (336.60–297.37)	33,7559 (30,5240–37,4221)	340.78 (307.85–376.34)	35,1526 (31,6618–39,1908)	341.00 (307.70–377.65)
Pakistan	34,8187 (30,6793–39,6906)	415.94 (364.40–474.79)	36,4964 (31,8033–41,6131)	199.91 (175.76–226.90)	34,9593 (30,6135–40,3560)	182.83 (161.59–208.49)
Nigeria	26,3238 (23,8491–29,1762)	389.24 (350.16–429.86)	29,9589 (26,6242–33,5459)	198.80 (175.49–223.15)	28,2068 (24,9958–31,8097)	178.16 (156.26–201.19)
Bangladesh	42,1817 (36,6614–48,0217)	524.87 (458.44–589.86)	23,2177 (20,2828–26,7288)	153.17 (133.92–175.81)	22,1100 (19,2226–25,6858)	140.67 (123.10–161.94)
DRC	16,2988 (14,4204–18,0504)	549.01 (494.13–607.15)	28,1284 (25,0834–31,4435)	441.16 (398.77–493.90)	28,2893 (25,1028–31,7333)	422.62 (377.90–470.73)

*DRC* Democratic Republic of the Congo, *ASIR* age-standardized incidence rate, *UI* uncertainty interval

### Effects of age, period and birth cohort on TB incidence for eight high-burden countries

**Fig. 2:**
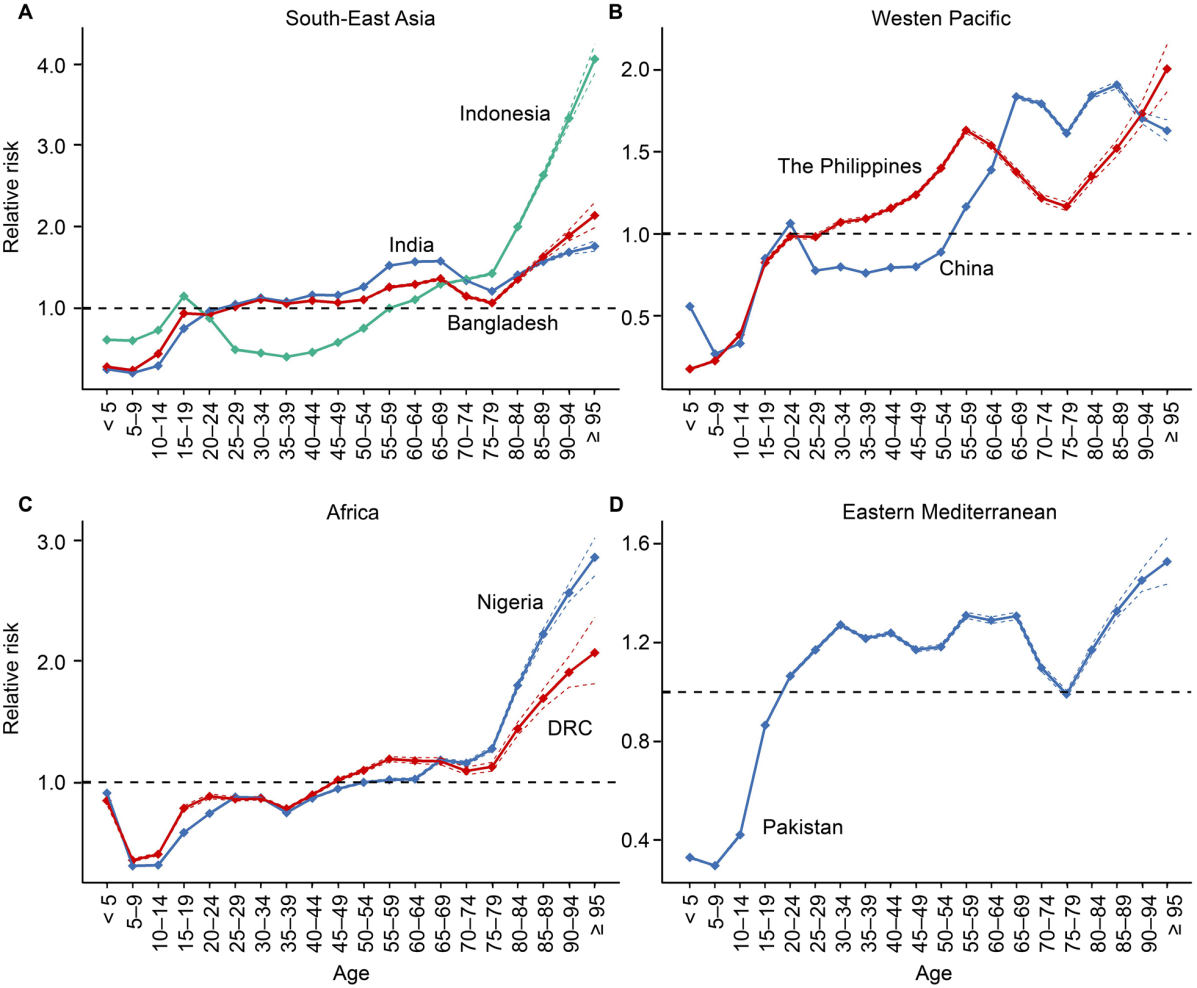
Age effect of tuberculosis in eight high-burden countries from 1990 to 2021. **A** High-burden tuberculosis countries in the Southeast Asia Region according to the World Health Organization. **B** High-burden tuberculosis countries in the Western Pacific Region according to the World Health Organization. **C** High-burden tuberculosis countries in the Africa Region according to the World Health Organization. **D** High-burden tuberculosis countries in the Eastern Mediterranean Region according to the World Health Organization. *DRC* the Democratic Republic of the Congo; the dotted lines indicate the 95% confidence interval

Regarding the age effect, all eight countries exhibited a high RR in older age groups, particularly those aged 75 and above. However, in India, a high RR was observed between the ages of 55 and 69. Indonesia showed a slight peak in the 15–19 age group (RR = 1.2, 95% *CI*: 1.1–1.2), while China exhibited a small peak between the ages of 20 and 24 (RR = 1.1, *P* < 0.001), with the RR remaining high from age 60 onwards. In the Philippines, the RR value for the 55–59 age group (RR = 1.6, 95% *CI*: 1.6–1.7) was higher compared to other age groups. In Pakistan, the risk of TB remained high from age 30 onwards. In Nigeria, RR values began exceeding 1 after age 55, while in Bangladesh, RR > 1 appeared earlier, at age 30. In the DRC, RR values were greater than 1 starting at age 50. It is worth noting that in India, the Philippines, Pakistan, and Bangladesh, the RR for TB incidence exceeded 1 after age 25. (Fig. 2A–D & Additional file 1: Table S2).

**Fig. 3:**
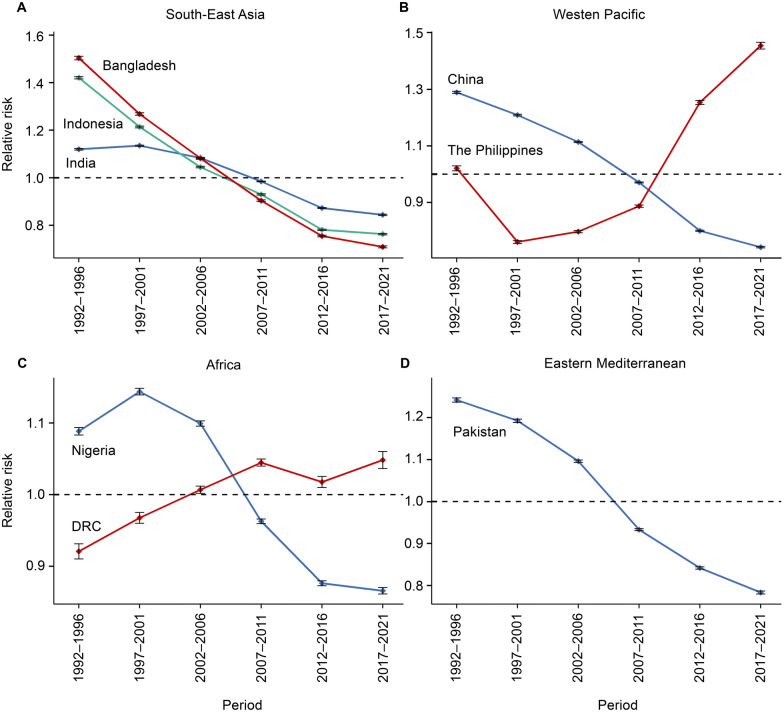
Period effect of tuberculosis in eight high-burden countries from 1990 to 2021. **A** High-burden tuberculosis countries in the Southeast Asia Region according to the World Health Organization. **B** High-burden tuberculosis countries in the Western Pacific Region according to the World Health Organization. **C** High-burden tuberculosis countries in the Africa Region according to the World Health Organization. **D** High-burden tuberculosis countries in the Eastern Mediterranean Region according to the World Health Organization. *DRC* the Democratic Republic of the Congo; the error lines indicate the 95% confidence interval

Regarding period effects, the risk of TB has decreased over the past three decades in India, Indonesia, China, Pakistan, and Bangladesh. However, two countries exhibited notable trends. In the Philippines, the RR has steadily increased since 2001, peaking in 2017–2021 (RR = 1.5, 95% *CI*: 1.44–1.47). In the DRC, the risk of TB has steadily risen over three decades, with a brief decline during 2012–2016, followed by a continuous increase, reaching its maximum in 2017–2021 (RR = 1.1, 95% *CI*: 1.0–1.1). (Fig. 3A–D & Additional file 1: Table S3).

**Fig. 4:**
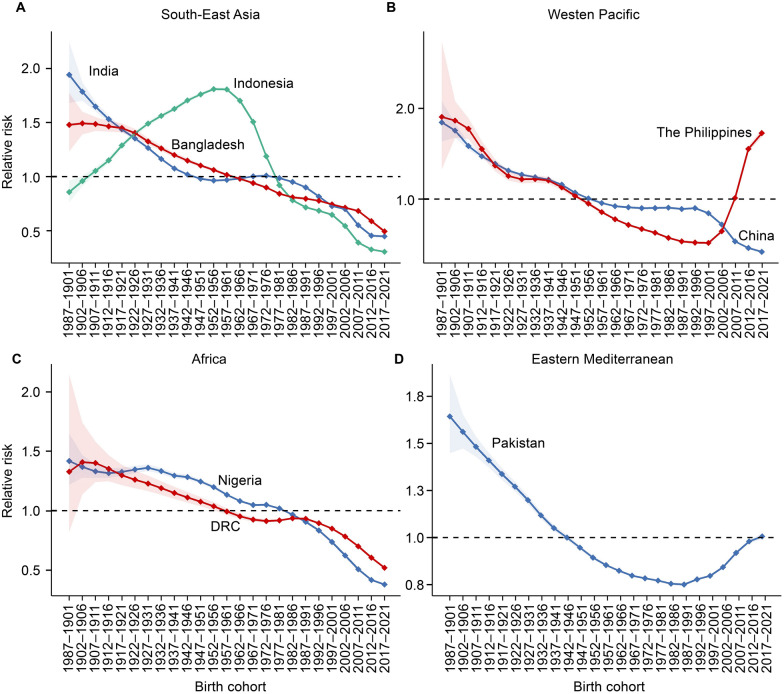
Cohort effect of tuberculosis in eight high-burden countries from 1990 to 2021. **A** High-burden tuberculosis countries in the Southeast Asia Region according to the World Health Organization. **B** High-burden tuberculosis countries in the Western Pacific Region according to the World Health Organization. **C** High-burden tuberculosis countries in the Africa Region according to the World Health Organization. **D** High-burden tuberculosis countries in the Eastern Mediterranean Region according to the World Health Organization. *DRC* the Democratic Republic of the Congo; the dashed areas indicate the 95% confidence interval

In India, China, Nigeria, Bangladesh, and the DRC, the RR of TB decreased with advancing birth cohorts, indicating that individuals born earlier were more likely to developing the TB compared to those born later. In Indonesia, the RR of TB initially increased and then decreased, with the highest RR observed in individuals born between 1952 and 1956 (RR = 1.8, *P* < 0.001). The Philippines exhibited a different trend, with the RR of TB initially decreasing and then increasing. The lowest RR was observed in individuals born between 1997 and 2001 (RR = 0.5, *P* < 0.001), after which the RR consistently rose, reaching as high as 1.7 (95% *CI*: 1.7–1.8) in individuals born between 2017 and 2021. Pakistan displayed a similar trend, with the lowest RR observed during 1987–1991(RR = 0.8, 95% *CI*: 0.8–0.8), followed by a gradual upward trend until 2021(Fig. 4A–D & Additional file 1: Table S4).

### Prediction to 2030 on TB incidence for eight high-burden countries

**Fig. 5:**
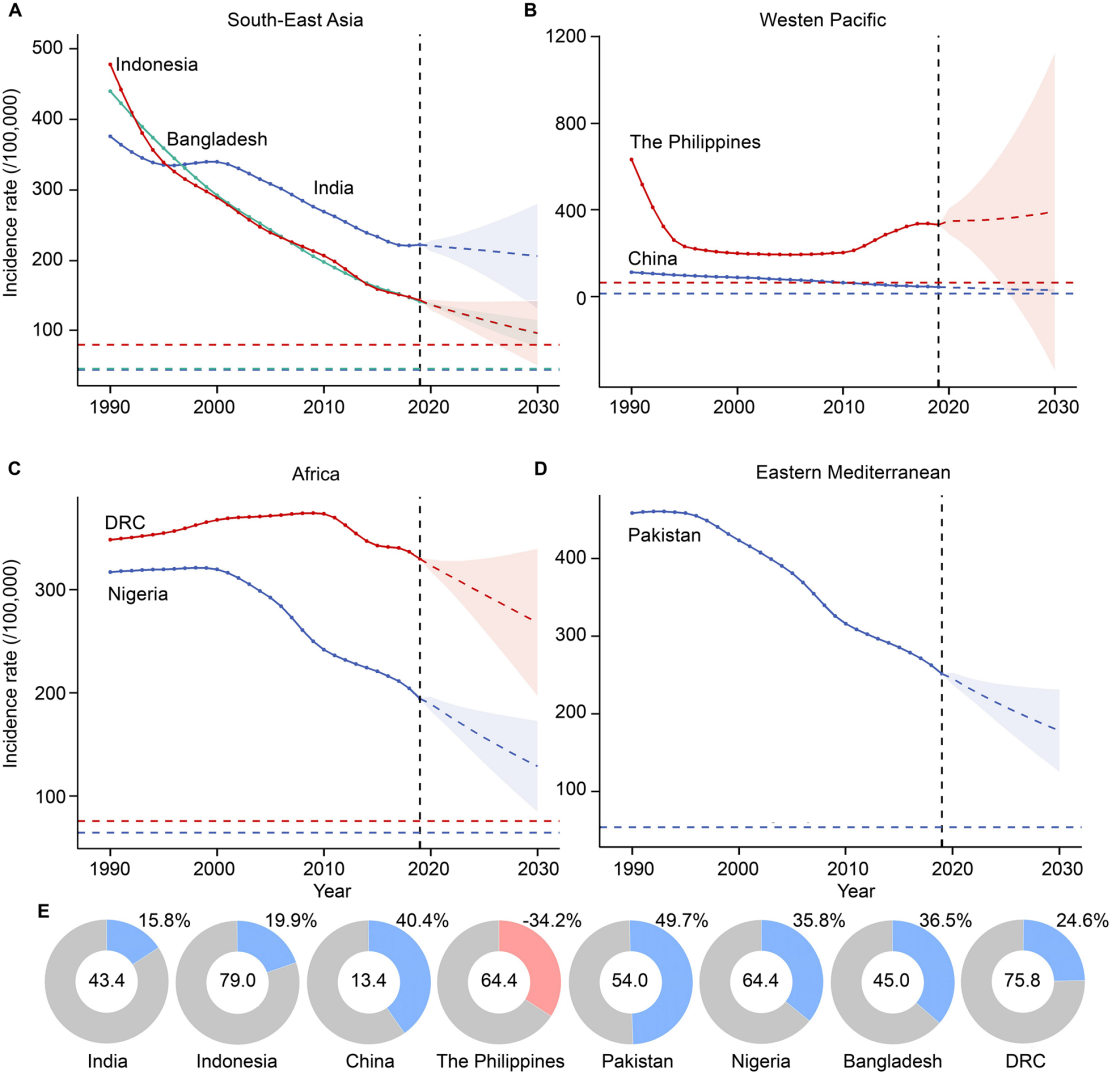
Prediction of tuberculosis in eight high-burden countries. **A** High-burden tuberculosis countries in the Southeast Asia Region according to the World Health Organization. **B** High-burden tuberculosis countries in the Western Pacific Region according to the World Health Organization. **C** High-burden tuberculosis countries in the Africa Region according to the World Health Organization. **D** High-burden tuberculosis countries in the Eastern Mediterranean Region according to the World Health Organization **E** Target completion ratio of eight high-burden countries. In **A**–**D** the colored dashed lines parallel to the X-axis represent the tuberculosis control targets for the corresponding countries. In **E** the numbers within the circles indicate the incidence rate of tuberculosis relative to the control targets, and the numbers in the upper right corner of the circles show the current level of progress, blue indicates a decrease in incidence, while red indicates an increase in incidence. *DRC* the Democratic Republic of the Congo; the dashed areas indicate the 95% confidence interval

Based on the forecast results, the ASIR of TB is projected to increase to 392.90/100,000 by 2030 in the Philippines, indicating an upward trend. In contrast, the rates have decreased in other countries, including India (205.41/100,000), Indonesia (95.84/100,000), China (29.03/100,000), Pakistan (178.46/100,000), Nigeria (128.86/100,000), Bangladesh (95.23/100,000), and the DRC (268.30/100,000). The study also quantitatively assessed the gaps that these eight countries need to address to achieve the WHO’s targets by 2030. The Philippines has the greatest gap from the target, with a completion rate of − 34.2%. Pakistan (49.7%) had the highest degree of completion, followed by China (40.4%), Bangladesh (36.5%), Nigeria (35.8%), the DRC (24.6%), Indonesia (19.9%), India (15.8%). (Fig. 5A–E & Additional file 1: Table S5).

## Discussion

The Global TB report 2023 indicated that two-thirds of global TB cases in 2022 were concentrated in eight countries: India, Indonesia, China, the Philippines, Pakistan, Nigeria, Bangladesh, and the DRC [4]. Achieving the WHO’s goal of eradicating TB requires effective control of its spread in these eight countries. This study aims to address this limitation and provide a comprehensive analysis of the TB situation in these high-burden countries using GBD 2021 data. It is important to note that although the GBD 2021 data used in this study employs different estimation methods compared to those of the WHO, the estimates are consistent [13].

The study found that young people in India, the Philippines, Pakistan, and Bangladesh are at a high risk of developing TB. Individuals in this age group play a crucial role in national development, societal progress, and family responsibilities. Their higher levels of activity compared to other age groups increase their exposure to TB patients, potentially leading to more frequent outbreaks within families, schools, and workplaces. Therefore, strengthening the management of TB patients in these countries is crucial, with identifying and screening individuals with TB from the general population being the top priority. However, the health resources available in Southeast Asia are insufficient for effectively managing TB cases [22]. There is an urgent need for a substantial increase in investment in TB prevention, diagnosis, care, and treatment to avoid the potentially disastrous consequences for the region. Additionally, this study revealed that TB incidence in China and Indonesia primarily affects the elderly population. These countries have a significant proportion of elderly individuals, and the aging trend has been steadily increasing in recent years [23, 24]. The demographic shift may be a crucial factor contributing to the higher incidence. Moreover, this study has identified a risk of TB among the younger age group (around 20 years old) in both countries, consistent with Zhe’s finding [7]. Therefore, TB prevention and control efforts in these countries should prioritize both adolescents and the elderly, with a focus on enhanced screening, prompt case detection, and early treatment. In contrast, our findings indicate that the incidence of TB in two African countries, Nigeria and the DRC, is notably high among children under 5 years old. This issue may be linked to insufficient vaccination efforts. Previous studies [25–27] highlight significant challenges in Africa’s vaccine production capacity, such as a shortage of trained personnel, inadequate public health infrastructure, limited access to maternal health facilities, unreliable information, and deep-rooted cultural beliefs that hinder vaccination efforts. Therefore, it is imperative for African leaders to actively promote vaccine development, while governments and the media should enhance public awareness and understanding of the benefits of vaccination. The second United Nations High-Level Meeting [28] on TB emphasized the longstanding neglect of children and adolescents by global and national policymakers, researchers, and manufacturers of TB diagnostic tools and treatments. It highlighted the urgent need to take decisive measures to mitigate the detrimental impact of TB on children and adolescents.

The ASIR and risk of TB has shown a downward trend over the past 30 years in most countries, although the rate of decline has slowed recently, potentially due to the impact of the COVID-19 pandemic. However, progress has fallen significantly short of the WHO targets, which aimed for a 20.0% reduction in TB incidence by 2020 compared to 2015 levels, and a 50.0% reduction by 2025. The setback caused by the COVID-19 pandemic has further complicated the achievement of these goals [2]. Notably, both the Philippines and the DRC have experienced an increasing trend in the incidence and RR of TB in recent years. The Philippines achieved nationwide coverage of directly observed treatment, short-course therapy in 2003. The national TB control program has become increasingly proactive. Since 2008, the management of drug-resistant TB has been integrated into the national TB plan [29, 30]. Since 2012, the implementation of the TB detection project in Palawan province has significantly improved TB reporting rate [31]. In the DRC, the National TB Control Center acquired GeneXpert devices in 2013, which have been widely used in rural areas. Since then, the number of detected TB cases has increased. Additionally, Rwanda established a TB/HIV collaboration mechanism at the central level through the Ministry of Public Health from 2004 to 2009, facilitating routine TB screening for HIV patients receiving antiretroviral therapy [32]. Furthermore, the Ebola virus disease outbreak in the DRC between 2018 and 2020 led to a misallocation of national health resources, resulting in the neglect of TB and contributing to a surge in new TB cases in recent years [33].

Cohort effects have revealed that the RR of developing TB was higher among individuals born before 1960 compared to those born later in eight high-burden TB countries. This suggested that screening, diagnosis, and treatment of TB should maintain vigilance, especially for older individuals. Among these countries, Indonesia and Nigeria also exhibited a higher risk of TB among middle-aged individuals (RR > 1.0), with smoking potentially being a contributing factor [34]. According to Indonesia’s basic health study data from 2018, 33.8% of individuals aged 15 and older were smokers. Co-infection or comorbidity with other pathogens or diseases, such as AIDS, diabetes and silicosis may also be significant factors, as these conditions are more prevalent among middle-aged and elderly individuals [35, 36]. Our research has also identified a concerning trend in the Philippines and Pakistan, where the risk of developing TB has gradually increased as more recent birth cohorts are considered since 1990. This indicates that individuals born in more recent years are becoming more susceptible to TB, particularly in the Philippines, where the RR has been greater than 1 since 2011. Previous studies have also highlighted that a significant proportion (27.3%) of TB patients in the Philippines fall within the age range of 0–24 years [37]. In Pakistan, approximately 369,000 TB cases were reported in 2018, with children accounting for 13.0% of these cases, accompanied by a high rate of under-reporting [38]. Several factors contribute to this phenomenon. First, individuals aged 10–24 years often spend prolonged periods in densely populated environments, such as schools, which greatly increases the risk of transmission. Additionally, there has been a notable increase in HIV infection rates among young people, with less than half of these individuals receiving antiretroviral therapy, thus significantly increasing their vulnerability to TB compared to those receiving treatment [35]. Half of the country’s population is under the age of 25, which is a significant factor in the increased risk of disease among young individuals [39]. Regarding TB control, other research [40, 41] has emphasized the need to focus not only on screening, diagnosis, and treatment but also on adopting a comprehensive grassroots approach that provides social protection to vulnerable groups and young individuals. Effective TB interventions in the Philippines should prioritize those most affected by the disease, including individuals living in overcrowded slums, economically disadvantaged populations lacking access to education and healthcare, individuals experiencing severe prison overcrowding, and young people living with HIV who do not have access to antiretroviral treatment. Additionally, underreporting of TB remains a significant concern in Pakistan. The childhood TB program implemented under the Zero TB initiative has been crucial in identifying unreported cases, and it is essential to sustain this program moving forward [42].

This study predicts that by 2030, the completion rate of the eight high-burden countries toward the WHO target will be less than 50.0%. Simultaneously, they are unlikely to meet the TB control targets set by their respective countries [7, 9, 22, 29, 32, 37]. Specifically, the incidence of TB in the Philippines is expected to continue to rising, while in India and Indonesia, the completion rate is projected to be less than 20.0%. Several factors affect the achievement of this goal, which this study analyzes as follows: gross domestic product (GDP) per capita and the prevalence of undernourishment are closely associated with TB incidence [2, 4]. In 2022, sub-Saharan Africa’s GDP (USD 2.1 trillion) and GDP per capita (USD 1701.2) were lower than the rest of the World Bank subregion [43]. According to the Food and Agriculture Organization [44], over 200 million individuals experienced undernourishment in sub-Saharan Africa during 2014–2016. The prevalence of undernourishment in this region rose from 181 million in 2010 to 222 million in 2016. Poverty is a primary cause of hunger and malnutrition in Africa, further contributing to the rise in diseases across the continent [45]. Furthermore, ambient air pollution is rising throughout Africa, posing significant threats to health, human capital, and economic development [46]. At the same time, the South Asia’s GDP was only slightly higher than that of sub-Saharan Africa [43], suggesting that the high levels in these regions are likely due to poverty and malnutrition. Therefore, effectively addressing issues such as malnutrition and exposure to air pollution may accelerate progress toward achieving these goals. An Indian study [47] revealed that, despite the availability of free diagnostic and treatment services under a national TB control program, households affected by TB faced a high risk of catastrophic costs and further impoverishment, highlighting the urgent need for additional financial protection for TB patients. A large population base is another challenge for effective TB control. According to the Chinese Bureau of Statistics [48], China’s population was projected to reach 1.4 billion in 2021, making it the largest in the world. Although China has made progress in reducing TB deaths and incidence rates over the past three decades [49], it is still far from meeting the targets set by the WHO’s End TB Strategy for 2030. It is worth emphasizing that China’s aging population is projected to continue accelerating. If effective control of TB among the elderly is not achieved, some of the aging countries, including China, may experience higher TB incidence than currently predicted.

This study has several limitations. Firstly, the data used in this study are based on GBD 2021, which provides estimated values. However, it is important to note that these estimates closely align with the figures reported by the WHO. Second, the study only performed a status analysis of the eight high-burden TB countries in the results section, without examining the risk factors. Third, the prediction model used in this study relies solely on time trends and does not account for the influence of multiple factors on the disease. Notably, environmental changes, the prevalence of other diseases, population aging, and updates to vaccines may all significantly impact forecast results; therefore, these results should be considered as preliminary references only.

## Conclusions

The study found that the RR of TB incidence decreases with age in India, the Philippines, Pakistan, and Bangladesh, while in Nigeria and the DRC, a higher RR was observed among children under five years old. TB incidence was also rising over time in the Philippines and the DRC. Additionally, there was a trend of increasing risk among more recent birth cohorts in the Philippines and Pakistan. Projections indicated that TB incidence in the Philippines is likely to continue rising until 2030, and completion rates for TB in India and Indonesia are expected to fall below 20% by then. This study highlights the need for tailored approaches to address the unique challenges faced by each country. It is crucial to promote neonatal vaccination and to enhance screening and management of TB patients in regions with limited health resources. Additionally, health assistance from international organizations is vital for countries with a high TB burden.

## Supplementary Information

Additional file 1.

## Data Availability

The data used were publicly for this study. The website of the data is: https://vizhub.healthdata.org/gbd-results/.

## Abbreviations

TB: Tuberculosis
COVID-19: Coronavirus disease 2019
HIV: Human immunodeficiency virus
AIDS: Acquired immune deficiency syndrome
WHO: World Health Organization
DRC: The Democratic Republic of the Congo
BAPC: Bayesian age-period-cohort
GBD: Global Burden of Diseases
DisMod-MR: Disease-model-Bayesian meta-regression
MI: Mortality-to-incidence
APC: Annual percentage change
AAPC: Average annual percentage change
*CI*: Confidence interval
IE: Intrinsic estimator
RR: Relative risk
ASIR: Age-standardized incidence rate
RW2: Second-order random walk
MCMC: Markov Chain Monte Carlo
