# Supplementary material for: Further analysis of tuberculosis in eight high-burden countries based on the Global Burden of Disease Study 2021 data
Source: Infect Dis Poverty. 2024 Sep 30;13:70. doi: 10.1186/s40249-024-01247-8 (PMC11440896; doi:10.1186/s40249-024-01247-8)
Supplement: Supplementary file 1 — Additional file 1. [file 40249_2024_1247_MOESM1_ESM.docx]

**Supplementary appendix**

Content

[Table S1. The analysis results of the incidence trends of tuberculosis among the eight high-burden countries from 1990–2021 1](#_Toc163897142)

[Table S2. The result of age effect of tuberculosis in eight high-burden countries 1](#_Toc163897143)

[Table S3. The result of period effect of tuberculosis in eight high-burden countries. 5](#_Toc163897144)

[Table S4. The result of cohort effect of tuberculosis in eight high-burden countries. 6](#_Toc163897145)

[Table S5. The forecast results of the incidence of tuberculosis in 8 high-burden countries(/100,000) 1](#_Toc163897146)2

| **Table S1. The temporal analysis results of the incidence of tuberculosis among the eight high-burden countries from 1990–2021** | | | | | | | | | | |
| --- | --- | --- | --- | --- | --- | --- | --- | --- | --- | --- |
| Country |  | Year | |  | APC/AAPC |  | 95% *CI* | |  | *P* |
|  |  | *lower* | *uppe*r |  |  |  | *lower* | *upper* |  |  |
| India |  | 1990 | 1995 |  | 0.39 |  | -0.36 | 1.15 |  | 0.293 |
|  |  | 1995 | 2016 |  | -2.84 |  | -2.91 | -2.76 |  | < 0.001 |
|  |  | 2016 | 2021 |  | -0.55 |  | -1.1 | -0.01 |  | 0.047 |
|  |  | 1990 | 2021 |  | -1.96 |  | -2.1 | -1.81 |  | < 0.001 |
| Indonesia |  | 1990 | 2021 |  | -2.21 |  | -2.33 | -2.08 |  | < 0.001 |
| China |  | 1990 | 2021 |  | -3.82 |  | -3.95 | -3.70 |  | < 0.001 |
| Philippines |  | 1990 | 2008 |  | -0.54 |  | -0.71 | -0.37 |  | < 0.001 |
|  |  | 2008 | 2021 |  | 3.05 |  | 2.76 | 3.33 |  | < 0.001 |
|  |  | 1990 | 2021 |  | 0.95 |  | 0.8 | 1.09 |  | < 0.001 |
| Pakistan |  | 1990 | 2000 |  | -0.74 |  | -0.97 | -0.50 |  | < 0.001 |
|  |  | 2000 | 2021 |  | -3.52 |  | -3.59 | -3.44 |  | < 0.001 |
| Nigeria |  | 1990 | 2002 |  | 0.70 |  | 0.53 | 0.87 |  | < 0.001 |
|  |  | 2002 | 2021 |  | -4.41 |  | -4.5 | -4.32 |  | < 0.001 |
|  |  | 1990 | 2021 |  | -2.46 |  | -2.54 | -2.38 |  | < 0.001 |
| Bangladesh |  | 1990 | 2021 |  | -4.17 |  | -4.24 | -4.10 |  | < 0.001 |
| DRC |  | 1990 | 1993 |  | -0.82 |  | -1.95 | 0.31 |  | 0.146 |
|  |  | 1993 | 2007 |  | 0.40 |  | 0.29 | 0.52 |  | < 0.001 |
|  |  | 2007 | 2021 |  | -2.00 |  | -2.1 | -1.90 |  | < 0.001 |
|  |  | 1990 | 2021 |  | -0.81 |  | -0.93 | -0.68 |  | < 0.001 |
| CI: confidence interval; APC: annual percentage change; AAPC: average annual percentage change; DRC: Democratic Republic of the Congo. | | | | | | | | | | |

| **Table S2: The result of age effect of tuberculosis in eight high-burden countries** | | | | | | | | | | |
| --- | --- | --- | --- | --- | --- | --- | --- | --- | --- | --- |
| Country |  | Group |  | Coefficient(95% *CI*) |  | RR(95% *CI*) |  | *Z* |  | *P* |
| India |  | Under 5 |  | -1.38(-1.40–-1.37) |  | 0.25(0.25–0.25) |  | -245.84 |  | <0.001 |
|  |  | 5–9 |  | -1.57(-1.58–-1.56) |  | 0.21(0.21–0.21) |  | -311.15 |  | <0.001 |
|  |  | 10–14 |  | -1.22(-1.23–-1.21) |  | 0.29(0.29–0.30) |  | -276.31 |  | <0.001 |
|  |  | 15–19 |  | -0.28(-0.29–-0.28) |  | 0.75(0.75–0.76) |  | -75.16 |  | <0.001 |
|  |  | 20–24 |  | -0.04(-0.04–-0.03) |  | 0.97(0.96–0.97) |  | -11.10 |  | <0.001 |
|  |  | 25–29 |  | 0.05(0.04–0.05) |  | 1.05(1.04–1.06) |  | 18.22 |  | <0.001 |
|  |  | 30–34 |  | 0.12(0.12–0.13) |  | 1.13(1.13–1.13) |  | 55.79 |  | <0.001 |
|  |  | 35–39 |  | 0.08(0.08–0.08) |  | 1.08(1.08–1.09) |  | 43.68 |  | <0.001 |
|  |  | 40–44 |  | 0.15(0.15–0.16) |  | 1.16(1.16–1.17) |  | 99.17 |  | <0.001 |
|  |  | 45–49 |  | 0.15(0.15–0.15) |  | 1.16(1.16–1.17) |  | 100.24 |  | <0.001 |
|  |  | 50–54 |  | 0.24(0.23–0.24) |  | 1.27(1.26–1.27) |  | 139.04 |  | <0.001 |
|  |  | 55–59 |  | 0.42(0.42–0.43) |  | 1.53(1.52–1.53) |  | 210.39 |  | <0.001 |
|  |  | 60–64 |  | 0.45(0.45–0.46) |  | 1.57(1.57–1.58) |  | 185.42 |  | <0.001 |
|  |  | 65–69 |  | 0.46(0.45–0.46) |  | 1.58(1.57–1.59) |  | 155.24 |  | <0.001 |
|  |  | 70–74 |  | 0.29(0.29–0.30) |  | 1.34(1.33–1.35) |  | 83.09 |  | <0.001 |
|  |  | 75–79 |  | 0.19(0.18–0.20) |  | 1.21(1.20–1.22) |  | 45.58 |  | <0.001 |
|  |  | 80–84 |  | 0.35(0.34–0.36) |  | 1.41(1.40–1.43) |  | 70.70 |  | <0.001 |
|  |  | 85–89 |  | 0.45(0.44–0.47) |  | 1.57(1.56–1.59) |  | 76.54 |  | <0.001 |
|  |  | 90–94 |  | 0.52(0.51–0.54) |  | 1.69(1.66–1.72) |  | 62.89 |  | <0.001 |
|  |  | 95 plus |  | 0.57(0.53–0.60) |  | 1.76(1.70–1.83) |  | 31.02 |  | <0.001 |
| Indonesia |  | Under 5 |  | -0.49(-0.50–-0.48) |  | 0.61(0.61–0.62) |  | -89.09 |  | <0.001 |
|  |  | 5–9 |  | -0.50(-0.51–-0.49) |  | 0.60(0.60–0.61) |  | -103.41 |  | <0.001 |
|  |  | 10–14 |  | -0.31(-0.32–-0.30) |  | 0.73(0.72–0.74) |  | -73.27 |  | <0.001 |
|  |  | 15–19 |  | 0.14(0.13–0.15) |  | 1.15(1.14–1.16) |  | 38.48 |  | <0.001 |
|  |  | 20–24 |  | -0.13(-0.14–-0.12) |  | 0.88(0.87–0.88) |  | -39.03 |  | <0.001 |
|  |  | 25–29 |  | -0.71(-0.71–-0.70) |  | 0.49(0.49–0.50) |  | -210.12 |  | <0.001 |
|  |  | 30–34 |  | -0.80(-0.80–-0.79) |  | 0.45(0.45–0.45) |  | -249.11 |  | <0.001 |
|  |  | 35–39 |  | -0.91(-0.91–-0.9) |  | 0.40(0.40–0.41) |  | -285.71 |  | <0.001 |
|  |  | 40–44 |  | -0.77(-0.78–-0.77) |  | 0.46(0.46–0.46) |  | -250.59 |  | <0.001 |
|  |  | 45–49 |  | -0.54(-0.55–-0.53) |  | 0.58(0.58–0.59) |  | -175.70 |  | <0.001 |
|  |  | 50–54 |  | -0.28(-0.28–-0.27) |  | 0.76(0.75–0.76) |  | -87.35 |  | <0.001 |
|  |  | 55–59 |  | 0.00(0.00–0.01) |  | 1.00(1.00–1.01) |  | 0.68 |  | 0.495 |
|  |  | 60–64 |  | 0.10(0.10–0.11) |  | 1.11(1.10–1.12) |  | 27.09 |  | <0.001 |
|  |  | 65–69 |  | 0.26(0.25–0.27) |  | 1.30(1.29–1.31) |  | 61.44 |  | <0.001 |
|  |  | 70–74 |  | 0.31(0.30–0.31) |  | 1.36(1.34–1.37) |  | 63.35 |  | <0.001 |
|  |  | 75–79 |  | 0.36(0.35–0.37) |  | 1.43(1.41–1.44) |  | 63.55 |  | <0.001 |
|  |  | 80–84 |  | 0.69(0.68–0.71) |  | 2.00(1.98–2.03) |  | 109.46 |  | <0.001 |
|  |  | 85–89 |  | 0.97(0.95–0.98) |  | 2.64(2.60–2.68) |  | 124.04 |  | <0.001 |
|  |  | 90–94 |  | 1.20(1.18–1.23) |  | 3.34(3.26–3.41) |  | 104.87 |  | <0.001 |
|  |  | 95 plus |  | 1.40(1.36–1.45) |  | 4.06(3.89–4.24) |  | 63.08 |  | <0.001 |
| China |  | Under 5 |  | -0.59(-0.60–-0.58) |  | 0.56(0.55–0.56) |  | -113.69 |  | <0.001 |
|  |  | 5–9 |  | -1.32(-1.33–-1.31) |  | 0.27(0.26–0.27) |  | -262.7 |  | <0.001 |
|  |  | 10–14 |  | -1.11(-1.12–-1.10) |  | 0.33(0.33–0.33) |  | -254.13 |  | <0.001 |
|  |  | 15–19 |  | -0.16(-0.17–-0.16) |  | 0.85(0.84–0.86) |  | -46.93 |  | <0.001 |
|  |  | 20–24 |  | 0.06(0.06–0.07) |  | 1.06(1.06–1.07) |  | 20.88 |  | <0.001 |
|  |  | 25–29 |  | -0.25(-0.26–-0.25) |  | 0.78(0.77–0.78) |  | -94.45 |  | <0.001 |
|  |  | 30–34 |  | -0.23(-0.23–-0.22) |  | 0.80(0.79–0.80) |  | -93.25 |  | <0.001 |
|  |  | 35–39 |  | -0.27(-0.28–-0.27) |  | 0.76(0.76–0.76) |  | -120.42 |  | <0.001 |
|  |  | 40–44 |  | -0.23(-0.23–-0.23) |  | 0.79(0.79–0.80) |  | -106.48 |  | <0.001 |
|  |  | 45–49 |  | -0.22(-0.23–-0.22) |  | 0.80(0.80–0.80) |  | -100.65 |  | <0.001 |
|  |  | 50–54 |  | -0.12(-0.12–-0.11) |  | 0.89(0.88–0.89) |  | -49.78 |  | <0.001 |
|  |  | 55–59 |  | 0.15(0.15–0.16) |  | 1.17(1.16–1.17) |  | 60.01 |  | <0.001 |
|  |  | 60–64 |  | 0.33(0.32–0.34) |  | 1.39(1.38–1.40) |  | 116.26 |  | <0.001 |
|  |  | 65–69 |  | 0.61(0.60–0.61) |  | 1.84(1.83–1.85) |  | 192.15 |  | <0.001 |
|  |  | 70–74 |  | 0.58(0.58–0.59) |  | 1.79(1.78–1.81) |  | 160.59 |  | <0.001 |
|  |  | 75–79 |  | 0.48(0.47–0.49) |  | 1.61(1.60–1.63) |  | 113.69 |  | <0.001 |
|  |  | 80–84 |  | 0.61(0.60–0.62) |  | 1.85(1.83–1.86) |  | 127.13 |  | <0.001 |
|  |  | 85–89 |  | 0.65(0.64–0.66) |  | 1.91(1.89–1.93) |  | 108.76 |  | <0.001 |
|  |  | 90–94 |  | 0.53(0.52–0.55) |  | 1.71(1.67–1.74) |  | 56.96 |  | <0.001 |
|  |  | 95 plus |  | 0.49(0.45–0.53) |  | 1.63(1.57–1.70) |  | 23.79 |  | <0.001 |
| Philippines |  | Under 5 |  | -1.75(-1.78–-1.72) |  | 0.17(0.17–0.18) |  | -116.49 |  | <0.001 |
|  |  | 5–9 |  | -1.49(-1.52–-1.47) |  | 0.22(0.22–0.23) |  | -112.77 |  | <0.001 |
|  |  | 10–14 |  | -0.96(-0.98–-0.93) |  | 0.38(0.38–0.39) |  | -82.51 |  | <0.001 |
|  |  | 15–19 |  | -0.19(-0.21–-0.17) |  | 0.82(0.81–0.84) |  | -19.54 |  | <0.001 |
|  |  | 20–24 |  | -0.01(-0.03–0.00) |  | 0.99(0.97–1.00) |  | -1.66 |  | 0.097 |
|  |  | 25–29 |  | -0.02(-0.03–0.00) |  | 0.98(0.97–1.00) |  | -2.34 |  | 0.019 |
|  |  | 30–34 |  | 0.07(0.06–0.08) |  | 1.07(1.06–1.08) |  | 10.84 |  | <0.001 |
|  |  | 35–39 |  | 0.09(0.08–0.10) |  | 1.09(1.08–1.11) |  | 16.62 |  | <0.001 |
|  |  | 40–44 |  | 0.15(0.14–0.16) |  | 1.16(1.15–1.17) |  | 30.2 |  | <0.001 |
|  |  | 45–49 |  | 0.21(0.20–0.22) |  | 1.24(1.23–1.25) |  | 45.22 |  | <0.001 |
|  |  | 50–54 |  | 0.34(0.33–0.35) |  | 1.40(1.39–1.42) |  | 67.21 |  | <0.001 |
|  |  | 55–59 |  | 0.49(0.48–0.50) |  | 1.63(1.62–1.65) |  | 86.34 |  | <0.001 |
|  |  | 60–64 |  | 0.43(0.42–0.45) |  | 1.54(1.52–1.56) |  | 63.7 |  | <0.001 |
|  |  | 65–69 |  | 0.32(0.30–0.34) |  | 1.38(1.36–1.40) |  | 39.3 |  | <0.001 |
|  |  | 70–74 |  | 0.20(0.18–0.22) |  | 1.22(1.20–1.24) |  | 20.25 |  | <0.001 |
|  |  | 75–79 |  | 0.16(0.13–0.18) |  | 1.17(1.14–1.19) |  | 13.52 |  | <0.001 |
|  |  | 80–84 |  | 0.30(0.28–0.33) |  | 1.35(1.32–1.39) |  | 22.71 |  | <0.001 |
|  |  | 85–89 |  | 0.42(0.39–0.45) |  | 1.52(1.48–1.57) |  | 26.34 |  | <0.001 |
|  |  | 90–94 |  | 0.55(0.51–0.59) |  | 1.73(1.66–1.81) |  | 25.95 |  | <0.001 |
|  |  | 95 plus |  | 0.70(0.62–0.77) |  | 2.01(1.87–2.16) |  | 18.96 |  | <0.001 |
| Pakistan |  | Under 5 |  | -1.10(-1.11–-1.09) |  | 0.33(0.33–0.34) |  | -194.54 |  | <0.001 |
|  |  | 5–9 |  | -1.21(-1.22–-1.20) |  | 0.30(0.30–0.30) |  | -237.2 |  | <0.001 |
|  |  | 10–14 |  | -0.86(-0.87–-0.85) |  | 0.42(0.42–0.43) |  | -194.15 |  | <0.001 |
|  |  | 15–19 |  | -0.14(-0.15–-0.14) |  | 0.87(0.86–0.87) |  | -38.91 |  | <0.001 |
|  |  | 20–24 |  | 0.06(0.06–0.07) |  | 1.07(1.06–1.07) |  | 19.55 |  | <0.001 |
|  |  | 25–29 |  | 0.16(0.15–0.16) |  | 1.17(1.16–1.18) |  | 52.98 |  | <0.001 |
|  |  | 30–34 |  | 0.24(0.23–0.25) |  | 1.27(1.26–1.28) |  | 83.62 |  | <0.001 |
|  |  | 35–39 |  | 0.20(0.19–0.20) |  | 1.22(1.21–1.22) |  | 65.1 |  | <0.001 |
|  |  | 40–44 |  | 0.21(0.21–0.22) |  | 1.24(1.23–1.25) |  | 65.83 |  | <0.001 |
|  |  | 45–49 |  | 0.16(0.15–0.17) |  | 1.17(1.16–1.18) |  | 43.01 |  | <0.001 |
|  |  | 50–54 |  | 0.17(0.16–0.18) |  | 1.18(1.17–1.19) |  | 40.54 |  | <0.001 |
|  |  | 55–59 |  | 0.27(0.26–0.28) |  | 1.31(1.30–1.32) |  | 58.6 |  | <0.001 |
|  |  | 60–64 |  | 0.25(0.24–0.26) |  | 1.29(1.28–1.30) |  | 49.06 |  | <0.001 |
|  |  | 65–69 |  | 0.27(0.26–0.28) |  | 1.31(1.29–1.32) |  | 46.44 |  | <0.001 |
|  |  | 70–74 |  | 0.09(0.08–0.11) |  | 1.10(1.08–1.11) |  | 14.22 |  | <0.001 |
|  |  | 75–79 |  | -0.01(-0.02–0.01) |  | 0.99(0.98–1.01) |  | -1.03 |  | 0.304 |
|  |  | 80–84 |  | 0.16(0.14–0.17) |  | 1.17(1.15–1.19) |  | 18.02 |  | <0.001 |
|  |  | 85–89 |  | 0.28(0.26–0.30) |  | 1.33(1.30–1.36) |  | 26.13 |  | <0.001 |
|  |  | 90–94 |  | 0.37(0.34–0.40) |  | 1.45(1.41–1.50) |  | 23.45 |  | <0.001 |
|  |  | 95 plus |  | 0.42(0.36–0.48) |  | 1.53(1.44–1.62) |  | 13.56 |  | <0.001 |
| Nigeria |  | Under 5 |  | -0.09(-0.11–-0.08) |  | 0.91(0.90–0.92) |  | -13.81 |  | <0.001 |
|  |  | 5–9 |  | -1.17(-1.18–-1.16) |  | 0.31(0.31–0.31) |  | -182.44 |  | <0.001 |
|  |  | 10–14 |  | -1.15(-1.16–-1.14) |  | 0.32(0.31–0.32) |  | -198.32 |  | <0.001 |
|  |  | 15–19 |  | -0.54(-0.54–-0.53) |  | 0.59(0.58–0.59) |  | -111.58 |  | <0.001 |
|  |  | 20–24 |  | -0.3(-0.30–-0.29) |  | 0.74(0.74–0.75) |  | -70.67 |  | <0.001 |
|  |  | 25–29 |  | -0.13(-0.14–-0.12) |  | 0.88(0.87–0.89) |  | -34.01 |  | <0.001 |
|  |  | 30–34 |  | -0.14(-0.14–-0.13) |  | 0.87(0.87–0.88) |  | -37.84 |  | <0.001 |
|  |  | 35–39 |  | -0.29(-0.30–-0.28) |  | 0.75(0.74–0.75) |  | -76.63 |  | <0.001 |
|  |  | 40–44 |  | -0.14(-0.15–-0.13) |  | 0.87(0.86–0.88) |  | -36.17 |  | <0.001 |
|  |  | 45–49 |  | -0.05(-0.06–-0.05) |  | 0.95(0.94–0.95) |  | -13.23 |  | <0.001 |
|  |  | 50–54 |  | 0.00(-0.01–0.01) |  | 1.00(0.99–1.01) |  | 0.27 |  | 0.785 |
|  |  | 55–59 |  | 0.02(0.01–0.03) |  | 1.02(1.01–1.03) |  | 4.15 |  | <0.001 |
|  |  | 60–64 |  | 0.03(0.02–0.04) |  | 1.03(1.02–1.04) |  | 4.58 |  | <0.001 |
|  |  | 65–69 |  | 0.17(0.16–0.18) |  | 1.18(1.17–1.20) |  | 26.98 |  | <0.001 |
|  |  | 70–74 |  | 0.15(0.13–0.16) |  | 1.16(1.14–1.17) |  | 20.77 |  | <0.001 |
|  |  | 75–79 |  | 0.25(0.23–0.26) |  | 1.28(1.26–1.30) |  | 30.99 |  | <0.001 |
|  |  | 80–84 |  | 0.59(0.57–0.61) |  | 1.80(1.77–1.83) |  | 66.94 |  | <0.001 |
|  |  | 85–89 |  | 0.80(0.78–0.82) |  | 2.23(2.18–2.27) |  | 74.48 |  | <0.001 |
|  |  | 90–94 |  | 0.94(0.91–0.97) |  | 2.57(2.49–2.64) |  | 62.05 |  | <0.001 |
|  |  | 95 plus |  | 1.05(1.00–1.11) |  | 2.86(2.71–3.02) |  | 37.43 |  | <0.001 |
| Bangladesh |  | Under 5 |  | -1.27(-1.28–-1.25) |  | 0.28(0.28–0.29) |  | -155.1 |  | <0.001 |
|  |  | 5–9 |  | -1.42(-1.44–-1.41) |  | 0.24(0.24–0.24) |  | -192.04 |  | <0.001 |
|  |  | 10–14 |  | -0.82(-0.83–-0.80) |  | 0.44(0.44–0.45) |  | -130.13 |  | <0.001 |
|  |  | 15–19 |  | -0.06(-0.07–-0.05) |  | 0.94(0.93–0.95) |  | -12.19 |  | <0.001 |
|  |  | 20–24 |  | -0.08(-0.09–-0.07) |  | 0.92(0.92–0.93) |  | -16.86 |  | <0.001 |
|  |  | 25–29 |  | 0.02(0.01–0.03) |  | 1.02(1.01–1.03) |  | 4.69 |  | <0.001 |
|  |  | 30–34 |  | 0.10(0.10–0.11) |  | 1.11(1.10–1.12) |  | 27.35 |  | <0.001 |
|  |  | 35–39 |  | 0.06(0.05–0.06) |  | 1.06(1.05–1.07) |  | 14.66 |  | <0.001 |
|  |  | 40–44 |  | 0.09(0.08–0.10) |  | 1.10(1.09–1.10) |  | 23.25 |  | <0.001 |
|  |  | 45–49 |  | 0.07(0.06–0.08) |  | 1.07(1.06–1.08) |  | 15.59 |  | <0.001 |
|  |  | 50–54 |  | 0.10(0.09–0.11) |  | 1.11(1.10–1.12) |  | 20.8 |  | <0.001 |
|  |  | 55–59 |  | 0.23(0.22–0.24) |  | 1.26(1.25–1.27) |  | 43.64 |  | <0.001 |
|  |  | 60–64 |  | 0.26(0.25–0.27) |  | 1.30(1.28–1.31) |  | 43.65 |  | <0.001 |
|  |  | 65–69 |  | 0.31(0.30–0.33) |  | 1.37(1.35–1.38) |  | 47.32 |  | <0.001 |
|  |  | 70–74 |  | 0.14(0.12–0.15) |  | 1.15(1.13–1.17) |  | 18.29 |  | <0.001 |
|  |  | 75–79 |  | 0.07(0.05–0.08) |  | 1.07(1.05–1.09) |  | 7.38 |  | <0.001 |
|  |  | 80–84 |  | 0.30(0.29–0.32) |  | 1.36(1.33–1.38) |  | 30.68 |  | <0.001 |
|  |  | 85–89 |  | 0.49(0.47–0.52) |  | 1.63(1.60–1.67) |  | 40.35 |  | <0.001 |
|  |  | 90–94 |  | 0.64(0.60–0.68) |  | 1.90(1.83–1.96) |  | 35.08 |  | <0.001 |
|  |  | 95 plus |  | 0.76(0.69–0.83) |  | 2.14(1.99–2.30) |  | 20.70 |  | <0.001 |
| DRC |  | Under 5 |  | -0.16(-0.20–-0.13) |  | 0.85(0.82–0.88) |  | -8.51 |  | <0.001 |
|  |  | 5–9 |  | -1.03(-1.06–-1.00) |  | 0.36(0.35–0.37) |  | -59.79 |  | <0.001 |
|  |  | 10–14 |  | -0.90(-0.93–-0.87) |  | 0.41(0.40–0.42) |  | -59.22 |  | <0.001 |
|  |  | 15–19 |  | -0.24(-0.27–-0.22) |  | 0.79(0.77–0.81) |  | -18.52 |  | <0.001 |
|  |  | 20–24 |  | -0.12(-0.14–-0.10) |  | 0.89(0.87–0.90) |  | -10.94 |  | <0.001 |
|  |  | 25–29 |  | -0.15(-0.17–-0.13) |  | 0.86(0.85–0.88) |  | -15.68 |  | <0.001 |
|  |  | 30–34 |  | -0.14(-0.16–-0.13) |  | 0.87(0.85–0.88) |  | -17.74 |  | <0.001 |
|  |  | 35–39 |  | -0.25(-0.26–-0.23) |  | 0.78(0.77–0.79) |  | -34.45 |  | <0.001 |
|  |  | 40–44 |  | -0.11(-0.12–-0.10) |  | 0.90(0.89–0.91) |  | -16.39 |  | <0.001 |
|  |  | 45–49 |  | 0.02(0.01–0.03) |  | 1.02(1.01–1.03) |  | 2.99 |  | 0.003 |
|  |  | 50–54 |  | 0.10(0.08–0.11) |  | 1.10(1.08–1.12) |  | 12.57 |  | <0.001 |
|  |  | 55–59 |  | 0.18(0.16–0.19) |  | 1.19(1.17–1.21) |  | 20.09 |  | <0.001 |
|  |  | 60–64 |  | 0.16(0.14–0.19) |  | 1.18(1.16–1.20) |  | 15.79 |  | <0.001 |
|  |  | 65–69 |  | 0.16(0.14–0.18) |  | 1.17(1.15–1.20) |  | 13.09 |  | <0.001 |
|  |  | 70–74 |  | 0.09(0.06–0.12) |  | 1.09(1.06–1.13) |  | 6.3 |  | <0.001 |
|  |  | 75–79 |  | 0.12(0.09–0.15) |  | 1.13(1.09–1.17) |  | 7.22 |  | <0.001 |
|  |  | 80–84 |  | 0.37(0.33–0.40) |  | 1.44(1.39–1.50) |  | 18.98 |  | <0.001 |
|  |  | 85–89 |  | 0.53(0.48–0.57) |  | 1.69(1.62–1.77) |  | 22.04 |  | <0.001 |
|  |  | 90–94 |  | 0.65(0.58–0.71) |  | 1.91(1.79–2.04) |  | 19 |  | <0.001 |
|  |  | 95 plus |  | 0.73(0.60–0.86) |  | 2.07(1.81–2.36) |  | 10.79 |  | <0.001 |
| DRC:Democratic Republic of the Congo; CI:confidential interval; RR:risk ratio | | | | | | | | | | |

| **Table S3: The result of period effect of tuberculosis in eight high-burden countries** | | | | | | | | | | |
| --- | --- | --- | --- | --- | --- | --- | --- | --- | --- | --- |
| Country |  | Group |  | Coefficient(95% *CI*) |  | RR(95% *CI*) |  | *Z* |  | *P* |
| India |  | 1992–1996 |  | 0.11(0.11–0.12) |  | 1.12(1.12–1.12) |  | 71.97 |  | <0.001 |
|  |  | 1997–2001 |  | 0.13(0.12–0.13) |  | 1.14(1.13–1.14) |  | 121.91 |  | <0.001 |
|  |  | 2002–2006 |  | 0.08(0.08–0.08) |  | 1.08(1.08–1.09) |  | 133.42 |  | <0.001 |
|  |  | 2007–2011 |  | -0.02(-0.02–-0.01) |  | 0.98(0.98–0.99) |  | -25.64 |  | <0.001 |
|  |  | 2012–2016 |  | -0.14(-0.14–-0.13) |  | 0.87(0.87–0.87) |  | -131.06 |  | <0.001 |
|  |  | 2017–2021 |  | -0.17(-0.17–-0.17) |  | 0.84(0.84–0.85) |  | -106.82 |  | <0.001 |
| Indonesia |  | 1992–1996 |  | 0.35(0.35–0.35) |  | 1.42(1.42–1.43) |  | 197.01 |  | <0.001 |
|  |  | 1997–2001 |  | 0.19(0.19–0.20) |  | 1.21(1.21–1.22) |  | 127.44 |  | <0.001 |
|  |  | 2002–2006 |  | 0.04(0.04–0.05) |  | 1.05(1.04–1.05) |  | 31.78 |  | <0.001 |
|  |  | 2007–2011 |  | -0.07(-0.08–-0.07) |  | 0.93(0.93–0.93) |  | -50.45 |  | <0.001 |
|  |  | 2012–2016 |  | -0.25(-0.25–-0.24) |  | 0.78(0.78–0.78) |  | -145.04 |  | <0.001 |
|  |  | 2017–2021 |  | -0.27(-0.27–-0.27) |  | 0.76(0.76–0.77) |  | -131.19 |  | <0.001 |
| China |  | 1992–1996 |  | 0.25(0.25–0.26) |  | 1.29(1.29–1.29) |  | 166.03 |  | <0.001 |
|  |  | 1997–2001 |  | 0.19(0.19–0.19) |  | 1.21(1.21–1.21) |  | 160.9 |  | <0.001 |
|  |  | 2002–2006 |  | 0.11(0.11–0.11) |  | 1.11(1.11–1.12) |  | 114.32 |  | <0.001 |
|  |  | 2007–2011 |  | -0.03(-0.03–-0.03) |  | 0.97(0.97–0.97) |  | -30.75 |  | <0.001 |
|  |  | 2012–2016 |  | -0.22(-0.23–-0.22) |  | 0.80(0.80–0.80) |  | -177.53 |  | <0.001 |
|  |  | 2017–2021 |  | -0.3(-0.30–-0.30) |  | 0.74(0.74–0.74) |  | -180.35 |  | <0.001 |
| Philippines |  | 1992–1996 |  | 0.02(0.01–0.03) |  | 1.02(1.01–1.03) |  | 4.76 |  | <0.001 |
|  |  | 1997–2001 |  | -0.27(-0.28–-0.27) |  | 0.76(0.76–0.77) |  | -80.4 |  | <0.001 |
|  |  | 2002–2006 |  | -0.23(-0.23–-0.22) |  | 0.80(0.79–0.80) |  | -87.84 |  | <0.001 |
|  |  | 2007–2011 |  | -0.12(-0.12–-0.12) |  | 0.89(0.88–0.89) |  | -50.14 |  | <0.001 |
|  |  | 2012–2016 |  | 0.23(0.22–0.23) |  | 1.25(1.25–1.26) |  | 77.22 |  | <0.001 |
|  |  | 2017–2021 |  | 0.37(0.37–0.38) |  | 1.45(1.44–1.47) |  | 90.48 |  | <0.001 |
| Pakistan |  | 1992–1996 |  | 0.22(0.21–0.22) |  | 1.24(1.24–1.25) |  | 103.53 |  | <0.001 |
|  |  | 1997–2001 |  | 0.18(0.17–0.18) |  | 1.19(1.19–1.20) |  | 104.88 |  | <0.001 |
|  |  | 2002–2006 |  | 0.09(0.09–0.09) |  | 1.10(1.09–1.10) |  | 67.92 |  | <0.001 |
|  |  | 2007–2011 |  | -0.07(-0.07–-0.07) |  | 0.93(0.93–0.94) |  | -51.50 |  | <0.001 |
|  |  | 2012–2016 |  | -0.17(-0.18–-0.17) |  | 0.84(0.84–0.84) |  | -104.47 |  | <0.001 |
|  |  | 2017–2021 |  | -0.24(-0.25–-0.24) |  | 0.78(0.78–0.79) |  | -110.50 |  | <0.001 |
| Nigeria |  | 1992–1996 |  | 0.08(0.08–0.09) |  | 1.09(1.08–1.09) |  | 33.88 |  | <0.001 |
|  |  | 1997–2001 |  | 0.13(0.13–0.14) |  | 1.14(1.14–1.15) |  | 66.37 |  | <0.001 |
|  |  | 2002–2006 |  | 0.09(0.09–0.10) |  | 1.10(1.10–1.10) |  | 55.72 |  | <0.001 |
|  |  | 2007–2011 |  | -0.04(-0.04–-0.03) |  | 0.96(0.96–0.97) |  | -22.00 |  | <0.001 |
|  |  | 2012–2016 |  | -0.13(-0.14–-0.13) |  | 0.88(0.87–0.88) |  | -64.77 |  | <0.001 |
|  |  | 2017–2021 |  | -0.14(-0.15–-0.14) |  | 0.87(0.86–0.87) |  | -55.52 |  | <0.001 |
| Bangladesh |  | 1992–1996 |  | 0.41(0.40–0.41) |  | 1.50(1.50–1.51) |  | 156.61 |  | <0.001 |
|  |  | 1997–2001 |  | 0.24(0.23–0.24) |  | 1.27(1.26–1.27) |  | 113.56 |  | <0.001 |
|  |  | 2002–2006 |  | 0.08(0.08–0.08) |  | 1.08(1.08–1.09) |  | 44.75 |  | <0.001 |
|  |  | 2007–2011 |  | -0.10(-0.10–-0.10) |  | 0.90(0.90–0.91) |  | -55.41 |  | <0.001 |
|  |  | 2012–2016 |  | -0.28(-0.28–-0.28) |  | 0.76(0.75–0.76) |  | -124.54 |  | <0.001 |
|  |  | 2017–2021 |  | -0.34(-0.35–-0.34) |  | 0.71(0.71–0.71) |  | -119.18 |  | <0.001 |
| DRC |  | 1992–1996 |  | -0.08(-0.09–-0.07) |  | 0.92(0.91–0.93) |  | -14.08 |  | <0.001 |
|  |  | 1997–2001 |  | -0.03(-0.04–-0.03) |  | 0.97(0.96–0.98) |  | -8.15 |  | <0.001 |
|  |  | 2002–2006 |  | 0.01(0.00–0.01) |  | 1.01(1.00–1.01) |  | 2.71 |  | 0.007 |
|  |  | 2007–2011 |  | 0.04(0.04–0.05) |  | 1.04(1.04–1.05) |  | 18.15 |  | <0.001 |
|  |  | 2012–2016 |  | 0.02(0.01–0.03) |  | 1.02(1.01–1.03) |  | 4.59 |  | <0.001 |
|  |  | 2017–2021 |  | 0.05(0.04–0.06) |  | 1.05(1.04–1.06) |  | 8.16 |  | <0.001 |
| DRC:APC:Democratic Republic of the Congo;age-perod-cohort;CI:confidential interval; RR:risk ratio | | | | | | | | | | |

| **Table S4: The result of cohort effect of tuberculosis in eight high-burden countries** | | | | | | | | | | |
| --- | --- | --- | --- | --- | --- | --- | --- | --- | --- | --- |
| Country |  | Group |  | Coefficient(95% *CI*) |  | RR(95% *CI*) |  | *Z* |  | *P* |
| India |  | 1897–1901 |  | 0.66(0.52–0.81) |  | 1.94(1.68–2.24) |  | 9.02 |  | <0.001 |
|  |  | 1902–1906 |  | 0.58(0.53–0.63) |  | 1.79(1.70–1.87) |  | 23.84 |  | <0.001 |
|  |  | 1907–1911 |  | 0.50(0.47–0.52) |  | 1.65(1.61–1.69) |  | 38.2 |  | <0.001 |
|  |  | 1912–1916 |  | 0.43(0.41–0.45) |  | 1.53(1.50–1.56) |  | 41.54 |  | <0.001 |
|  |  | 1917–1921 |  | 0.36(0.34–0.38) |  | 1.44(1.41–1.46) |  | 40.4 |  | <0.001 |
|  |  | 1922–1926 |  | 0.30(0.29–0.32) |  | 1.35(1.33–1.37) |  | 37.56 |  | <0.001 |
|  |  | 1927–1931 |  | 0.24(0.22–0.25) |  | 1.27(1.25–1.28) |  | 32.02 |  | <0.001 |
|  |  | 1932–1936 |  | 0.15(0.14–0.16) |  | 1.16(1.15–1.18) |  | 22.51 |  | <0.001 |
|  |  | 1937–1941 |  | 0.07(0.06–0.08) |  | 1.08(1.06–1.09) |  | 11.89 |  | <0.001 |
|  |  | 1942–1946 |  | 0.02(0.01–0.03) |  | 1.02(1.01–1.03) |  | 3.62 |  | <0.001 |
|  |  | 1947–1951 |  | -0.02(-0.03–-0.01) |  | 0.98(0.97–0.99) |  | -3.91 |  | <0.001 |
|  |  | 1952–1956 |  | -0.04(-0.04–-0.03) |  | 0.96(0.96–0.97) |  | -8.3 |  | <0.001 |
|  |  | 1957–1961 |  | -0.03(-0.04–-0.02) |  | 0.97(0.96–0.98) |  | -8.14 |  | <0.001 |
|  |  | 1962–1966 |  | -0.02(-0.02–-0.01) |  | 0.98(0.98–0.99) |  | -5.31 |  | <0.001 |
|  |  | 1967–1971 |  | 0.00(0.00–0.01) |  | 1.00(1.00–1.01) |  | 1.12 |  | 0.261 |
|  |  | 1972–1976 |  | 0.01(0.01–0.01) |  | 1.01(1.01–1.01) |  | 4.84 |  | <0.001 |
|  |  | 1977–1981 |  | -0.01(-0.02–-0.01) |  | 0.99(0.98–0.99) |  | -8.3 |  | <0.001 |
|  |  | 1982–1986 |  | -0.05(-0.05–-0.05) |  | 0.95(0.95–0.95) |  | -41.16 |  | <0.001 |
|  |  | 1987–1991 |  | -0.10(-0.11–-0.10) |  | 0.90(0.90–0.90) |  | -99.94 |  | <0.001 |
|  |  | 1992–1996 |  | -0.20(-0.21–-0.20) |  | 0.82(0.81–0.82) |  | -162.94 |  | <0.001 |
|  |  | 1997–2001 |  | -0.32(-0.32–-0.31) |  | 0.73(0.73–0.73) |  | -181.36 |  | <0.001 |
|  |  | 2002–2006 |  | -0.36(-0.36–-0.35) |  | 0.70(0.70–0.70) |  | -148.96 |  | <0.001 |
|  |  | 2007–2011 |  | -0.59(-0.60–-0.59) |  | 0.55(0.55–0.56) |  | -175.6 |  | <0.001 |
|  |  | 2012–2016 |  | -0.79(-0.8–-0.78) |  | 0.46(0.45–0.46) |  | -172.84 |  | <0.001 |
|  |  | 2017–2021 |  | -0.80(-0.81–-0.79) |  | 0.45(0.44–0.45) |  | -126.71 |  | <0.001 |
| Indonesia |  | 1897–1901 |  | -0.15(-0.27–-0.04) |  | 0.86(0.77–0.96) |  | -2.63 |  | 0.009 |
|  |  | 1902–1906 |  | -0.04(-0.09–0.01) |  | 0.96(0.91–1.01) |  | -1.65 |  | 0.099 |
|  |  | 1907–1911 |  | 0.05(0.02–0.08) |  | 1.05(1.02–1.09) |  | 3.28 |  | 0.001 |
|  |  | 1912–1916 |  | 0.14(0.12–0.16) |  | 1.15(1.12–1.18) |  | 11.87 |  | <0.001 |
|  |  | 1917–1921 |  | 0.25(0.23–0.27) |  | 1.29(1.26–1.31) |  | 25.93 |  | <0.001 |
|  |  | 1922–1926 |  | 0.34(0.32–0.35) |  | 1.40(1.38–1.42) |  | 40.22 |  | <0.001 |
|  |  | 1927–1931 |  | 0.40(0.38–0.41) |  | 1.49(1.47–1.51) |  | 53.51 |  | <0.001 |
|  |  | 1932–1936 |  | 0.45(0.43–0.46) |  | 1.56(1.54–1.58) |  | 65.65 |  | <0.001 |
|  |  | 1937–1941 |  | 0.49(0.47–0.50) |  | 1.63(1.61–1.65) |  | 78.19 |  | <0.001 |
|  |  | 1942–1946 |  | 0.53(0.52–0.54) |  | 1.70(1.69–1.72) |  | 93.27 |  | <0.001 |
|  |  | 1947–1951 |  | 0.57(0.56–0.58) |  | 1.76(1.74–1.78) |  | 108.02 |  | <0.001 |
|  |  | 1952–1956 |  | 0.59(0.58–0.60) |  | 1.81(1.79–1.83) |  | 124.82 |  | <0.001 |
|  |  | 1957–1961 |  | 0.59(0.58–0.60) |  | 1.81(1.79–1.82) |  | 137.65 |  | <0.001 |
|  |  | 1962–1966 |  | 0.53(0.52–0.54) |  | 1.70(1.69–1.72) |  | 136.79 |  | <0.001 |
|  |  | 1967–1971 |  | 0.41(0.40–0.42) |  | 1.51(1.50–1.52) |  | 115.31 |  | <0.001 |
|  |  | 1972–1976 |  | 0.17(0.17–0.18) |  | 1.19(1.18–1.20) |  | 54.22 |  | <0.001 |
|  |  | 1977–1981 |  | -0.08(-0.09–-0.08) |  | 0.92(0.92–0.93) |  | -28.95 |  | <0.001 |
|  |  | 1982–1986 |  | -0.25(-0.25–-0.24) |  | 0.78(0.78–0.79) |  | -91.88 |  | <0.001 |
|  |  | 1987–1991 |  | -0.33(-0.34–-0.33) |  | 0.72(0.71–0.72) |  | -126.35 |  | <0.001 |
|  |  | 1992–1996 |  | -0.38(-0.38–-0.37) |  | 0.69(0.68–0.69) |  | -141.95 |  | <0.001 |
|  |  | 1997–2001 |  | -0.43(-0.44–-0.43) |  | 0.65(0.64–0.65) |  | -140.65 |  | <0.001 |
|  |  | 2002–2006 |  | -0.61(-0.61–-0.60) |  | 0.54(0.54–0.55) |  | -157.66 |  | <0.001 |
|  |  | 2007–2011 |  | -0.94(-0.95–-0.93) |  | 0.39(0.39–0.39) |  | -172.4 |  | <0.001 |
|  |  | 2012–2016 |  | -1.12(-1.13–-1.10) |  | 0.33(0.32–0.33) |  | -147.82 |  | <0.001 |
|  |  | 2017–2021 |  | -1.18(-1.21–-1.16) |  | 0.31(0.30–0.31) |  | -99.07 |  | <0.001 |
| China |  | 1897–1901 |  | 0.61(0.49–0.73) |  | 1.85(1.64–2.08) |  | 10 |  | <0.001 |
|  |  | 1902–1906 |  | 0.56(0.52–0.61) |  | 1.76(1.68–1.84) |  | 24.5 |  | <0.001 |
|  |  | 1907–1911 |  | 0.46(0.44–0.48) |  | 1.59(1.55–1.62) |  | 37.45 |  | <0.001 |
|  |  | 1912–1916 |  | 0.39(0.37–0.40) |  | 1.47(1.45–1.50) |  | 41.74 |  | <0.001 |
|  |  | 1917–1921 |  | 0.33(0.31–0.35) |  | 1.39(1.37–1.41) |  | 41.2 |  | <0.001 |
|  |  | 1922–1926 |  | 0.27(0.26–0.29) |  | 1.32(1.30–1.33) |  | 38.16 |  | <0.001 |
|  |  | 1927–1931 |  | 0.24(0.23–0.25) |  | 1.27(1.25–1.29) |  | 36.41 |  | <0.001 |
|  |  | 1932–1936 |  | 0.22(0.21–0.23) |  | 1.24(1.23–1.26) |  | 36.12 |  | <0.001 |
|  |  | 1937–1941 |  | 0.20(0.18–0.21) |  | 1.22(1.20–1.23) |  | 35.47 |  | <0.001 |
|  |  | 1942–1946 |  | 0.15(0.14–0.16) |  | 1.16(1.15–1.17) |  | 29.4 |  | <0.001 |
|  |  | 1947–1951 |  | 0.07(0.06–0.08) |  | 1.07(1.06–1.08) |  | 15.11 |  | <0.001 |
|  |  | 1952–1956 |  | 0.01(0.00–0.02) |  | 1.01(1.00–1.02) |  | 1.91 |  | 0.056 |
|  |  | 1957–1961 |  | -0.04(-0.05–-0.04) |  | 0.96(0.95–0.96) |  | -11.96 |  | <0.001 |
|  |  | 1962–1966 |  | -0.08(-0.09–-0.07) |  | 0.92(0.92–0.93) |  | -24.73 |  | <0.001 |
|  |  | 1967–1971 |  | -0.09(-0.10–-0.09) |  | 0.91(0.91–0.92) |  | -33.4 |  | <0.001 |
|  |  | 1972–1976 |  | -0.10(-0.11–-0.10) |  | 0.90(0.90–0.91) |  | -43.07 |  | <0.001 |
|  |  | 1977–1981 |  | -0.10(-0.11–-0.10) |  | 0.90(0.90–0.91) |  | -47.25 |  | <0.001 |
|  |  | 1982–1986 |  | -0.10(-0.10–-0.09) |  | 0.91(0.90–0.91) |  | -47.36 |  | <0.001 |
|  |  | 1987–1991 |  | -0.11(-0.12–-0.11) |  | 0.89(0.89–0.90) |  | -57.63 |  | <0.001 |
|  |  | 1992–1996 |  | -0.10(-0.11–-0.10) |  | 0.90(0.90–0.91) |  | -49.88 |  | <0.001 |
|  |  | 1997–2001 |  | -0.17(-0.17–-0.16) |  | 0.84(0.84–0.85) |  | -61.65 |  | <0.001 |
|  |  | 2002–2006 |  | -0.33(-0.33–-0.32) |  | 0.72(0.72–0.73) |  | -86.28 |  | <0.001 |
|  |  | 2007–2011 |  | -0.63(-0.64–-0.61) |  | 0.53(0.53–0.54) |  | -110.33 |  | <0.001 |
|  |  | 2012–2016 |  | -0.77(-0.78–-0.76) |  | 0.46(0.46–0.47) |  | -105.41 |  | <0.001 |
|  |  | 2017–2021 |  | -0.87(-0.89–-0.85) |  | 0.42(0.41–0.43) |  | -89.47 |  | <0.001 |
| Philippines |  | 1897–1901 |  | 0.64(0.29–1.00) |  | 1.91(1.33–2.73) |  | 3.53 |  | <0.001 |
|  |  | 1902–1906 |  | 0.62(0.52–0.73) |  | 1.87(1.68–2.08) |  | 11.36 |  | <0.001 |
|  |  | 1907–1911 |  | 0.57(0.51–0.64) |  | 1.77(1.66–1.89) |  | 17.35 |  | <0.001 |
|  |  | 1912–1916 |  | 0.44(0.39–0.49) |  | 1.55(1.47–1.64) |  | 15.93 |  | <0.001 |
|  |  | 1917–1921 |  | 0.32(0.27–0.36) |  | 1.37(1.31–1.44) |  | 13 |  | <0.001 |
|  |  | 1922–1926 |  | 0.23(0.19–0.27) |  | 1.26(1.20–1.31) |  | 10.6 |  | <0.001 |
|  |  | 1927–1931 |  | 0.20(0.16–0.24) |  | 1.22(1.17–1.27) |  | 10.27 |  | <0.001 |
|  |  | 1932–1936 |  | 0.20(0.17–0.23) |  | 1.22(1.18–1.26) |  | 11.46 |  | <0.001 |
|  |  | 1937–1941 |  | 0.19(0.16–0.22) |  | 1.21(1.17–1.25) |  | 11.9 |  | <0.001 |
|  |  | 1942–1946 |  | 0.12(0.09–0.15) |  | 1.13(1.10–1.16) |  | 8.49 |  | <0.001 |
|  |  | 1947–1951 |  | 0.04(0.01–0.06) |  | 1.04(1.01–1.06) |  | 2.85 |  | 0.004 |
|  |  | 1952–1956 |  | -0.05(-0.07–-0.03) |  | 0.95(0.93–0.97) |  | -4.41 |  | <0.001 |
|  |  | 1957–1961 |  | -0.15(-0.17–-0.13) |  | 0.86(0.84–0.87) |  | -15.45 |  | <0.001 |
|  |  | 1962–1966 |  | -0.25(-0.27–-0.23) |  | 0.78(0.77–0.79) |  | -29.14 |  | <0.001 |
|  |  | 1967–1971 |  | -0.33(-0.35–-0.32) |  | 0.72(0.71–0.73) |  | -45.71 |  | <0.001 |
|  |  | 1972–1976 |  | -0.40(-0.41–-0.39) |  | 0.67(0.66–0.68) |  | -65.68 |  | <0.001 |
|  |  | 1977–1981 |  | -0.46(-0.47–-0.45) |  | 0.63(0.62–0.64) |  | -92.48 |  | <0.001 |
|  |  | 1982–1986 |  | -0.56(-0.56–-0.55) |  | 0.57(0.57–0.58) |  | -128.22 |  | <0.001 |
|  |  | 1987–1991 |  | -0.63(-0.63–-0.62) |  | 0.54(0.53–0.54) |  | -149.71 |  | <0.001 |
|  |  | 1992–1996 |  | -0.65(-0.66–-0.64) |  | 0.52(0.52–0.53) |  | -145.43 |  | <0.001 |
|  |  | 1997–2001 |  | -0.66(-0.67–-0.65) |  | 0.52(0.51–0.52) |  | -120.21 |  | <0.001 |
|  |  | 2002–2006 |  | -0.44(-0.45–-0.42) |  | 0.65(0.64–0.66) |  | -65.07 |  | <0.001 |
|  |  | 2007–2011 |  | 0.01(0.00–0.03) |  | 1.01(1.00–1.03) |  | 1.54 |  | 0.124 |
|  |  | 2012–2016 |  | 0.44(0.42–0.46) |  | 1.55(1.52–1.58) |  | 43.91 |  | <0.001 |
|  |  | 2017–2021 |  | 0.55(0.52–0.57) |  | 1.73(1.68–1.77) |  | 41.09 |  | <0.001 |
| Pakistan |  | 1897–1901 |  | 0.50(0.37–0.62) |  | 1.64(1.45–1.86) |  | 7.72 |  | <0.001 |
|  |  | 1902–1906 |  | 0.45(0.39–0.50) |  | 1.56(1.47–1.66) |  | 15.09 |  | <0.001 |
|  |  | 1907–1911 |  | 0.39(0.36–0.43) |  | 1.48(1.43–1.54) |  | 21.56 |  | <0.001 |
|  |  | 1912–1916 |  | 0.34(0.32–0.37) |  | 1.41(1.37–1.45) |  | 24.98 |  | <0.001 |
|  |  | 1917–1921 |  | 0.29(0.27–0.31) |  | 1.34(1.31–1.37) |  | 24.79 |  | <0.001 |
|  |  | 1922–1926 |  | 0.24(0.22–0.26) |  | 1.27(1.25–1.30) |  | 23.31 |  | <0.001 |
|  |  | 1927–1931 |  | 0.18(0.16–0.20) |  | 1.20(1.18–1.22) |  | 19.69 |  | <0.001 |
|  |  | 1932–1936 |  | 0.11(0.10–0.13) |  | 1.12(1.10–1.14) |  | 13.36 |  | <0.001 |
|  |  | 1937–1941 |  | 0.05(0.03–0.06) |  | 1.05(1.04–1.07) |  | 6.52 |  | <0.001 |
|  |  | 1942–1946 |  | 0.00(-0.01–0.01) |  | 1.00(0.99–1.01) |  | 0.06 |  | 0.951 |
|  |  | 1947–1951 |  | -0.05(-0.07–-0.04) |  | 0.95(0.94–0.96) |  | -8.7 |  | <0.001 |
|  |  | 1952–1956 |  | -0.11(-0.12–-0.10) |  | 0.89(0.88–0.90) |  | -19.96 |  | <0.001 |
|  |  | 1957–1961 |  | -0.16(-0.17–-0.15) |  | 0.85(0.85–0.86) |  | -31.83 |  | <0.001 |
|  |  | 1962–1966 |  | -0.19(-0.20–-0.18) |  | 0.82(0.82–0.83) |  | -44.92 |  | <0.001 |
|  |  | 1967–1971 |  | -0.23(-0.23–-0.22) |  | 0.80(0.79–0.8) |  | -61.39 |  | <0.001 |
|  |  | 1972–1976 |  | -0.24(-0.25–-0.24) |  | 0.78(0.78–0.79) |  | -79.36 |  | <0.001 |
|  |  | 1977–1981 |  | -0.26(-0.26–-0.25) |  | 0.77(0.77–0.78) |  | -101.67 |  | <0.001 |
|  |  | 1982–1986 |  | -0.28(-0.28–-0.28) |  | 0.76(0.75–0.76) |  | -124.36 |  | <0.001 |
|  |  | 1987–1991 |  | -0.29(-0.29–-0.28) |  | 0.75(0.75–0.75) |  | -130.62 |  | <0.001 |
|  |  | 1992–1996 |  | -0.25(-0.25–-0.25) |  | 0.78(0.78–0.78) |  | -107.87 |  | <0.001 |
|  |  | 1997–2001 |  | -0.23(-0.23–-0.22) |  | 0.80(0.79–0.80) |  | -80.47 |  | <0.001 |
|  |  | 2002–2006 |  | -0.17(-0.18–-0.16) |  | 0.84(0.84–0.85) |  | -48.59 |  | <0.001 |
|  |  | 2007–2011 |  | -0.08(-0.09–-0.08) |  | 0.92(0.91–0.93) |  | -19.09 |  | <0.001 |
|  |  | 2012–2016 |  | -0.02(-0.03–-0.01) |  | 0.98(0.97–0.99) |  | -3.68 |  | <0.001 |
|  |  | 2017–2021 |  | 0.01(-0.01–0.02) |  | 1.01(0.99–1.02) |  | 0.9 |  | 0.367 |
| Nigeria |  | 1897–1901 |  | 0.35(0.20–0.50) |  | 1.42(1.22–1.65) |  | 4.53 |  | <0.001 |
|  |  | 1902–1906 |  | 0.31(0.24–0.38) |  | 1.37(1.27–1.47) |  | 8.68 |  | <0.001 |
|  |  | 1907–1911 |  | 0.29(0.24–0.33) |  | 1.33(1.27–1.39) |  | 13.07 |  | <0.001 |
|  |  | 1912–1916 |  | 0.27(0.24–0.31) |  | 1.32(1.28–1.36) |  | 17.16 |  | <0.001 |
|  |  | 1917–1921 |  | 0.28(0.26–0.31) |  | 1.33(1.29–1.36) |  | 21.33 |  | <0.001 |
|  |  | 1922–1926 |  | 0.30(0.27–0.32) |  | 1.35(1.32–1.38) |  | 25.81 |  | <0.001 |
|  |  | 1927–1931 |  | 0.31(0.29–0.33) |  | 1.36(1.33–1.39) |  | 29.47 |  | <0.001 |
|  |  | 1932–1936 |  | 0.29(0.27–0.31) |  | 1.33(1.31–1.36) |  | 29.82 |  | <0.001 |
|  |  | 1937–1941 |  | 0.26(0.24–0.28) |  | 1.29(1.27–1.32) |  | 29.1 |  | <0.001 |
|  |  | 1942–1946 |  | 0.25(0.23–0.26) |  | 1.28(1.26–1.30) |  | 30.79 |  | <0.001 |
|  |  | 1947–1951 |  | 0.22(0.20–0.23) |  | 1.24(1.23–1.26) |  | 29.46 |  | <0.001 |
|  |  | 1952–1956 |  | 0.18(0.17–0.19) |  | 1.20(1.18–1.21) |  | 27.16 |  | <0.001 |
|  |  | 1957–1961 |  | 0.13(0.11–0.14) |  | 1.13(1.12–1.15) |  | 21 |  | <0.001 |
|  |  | 1962–1966 |  | 0.08(0.07–0.09) |  | 1.08(1.07–1.09) |  | 14.86 |  | <0.001 |
|  |  | 1967–1971 |  | 0.05(0.04–0.06) |  | 1.05(1.04–1.06) |  | 10.49 |  | <0.001 |
|  |  | 1972–1976 |  | 0.05(0.04–0.06) |  | 1.05(1.04–1.06) |  | 12.68 |  | <0.001 |
|  |  | 1977–1981 |  | 0.02(0.01–0.02) |  | 1.02(1.01–1.02) |  | 5.56 |  | <0.001 |
|  |  | 1982–1986 |  | -0.03(-0.04–-0.03) |  | 0.97(0.96–0.97) |  | -11.82 |  | <0.001 |
|  |  | 1987–1991 |  | -0.10(-0.10–-0.09) |  | 0.91(0.90–0.91) |  | -34.81 |  | <0.001 |
|  |  | 1992–1996 |  | -0.18(-0.19–-0.18) |  | 0.83(0.83–0.84) |  | -66.67 |  | <0.001 |
|  |  | 1997–2001 |  | -0.31(-0.31–-0.30) |  | 0.74(0.73–0.74) |  | -92.47 |  | <0.001 |
|  |  | 2002–2006 |  | -0.47(-0.48–-0.47) |  | 0.62(0.62–0.63) |  | -114.85 |  | <0.001 |
|  |  | 2007–2011 |  | -0.68(-0.69–-0.67) |  | 0.51(0.50–0.51) |  | -131.23 |  | <0.001 |
|  |  | 2012–2016 |  | -0.88(-0.89–-0.86) |  | 0.42(0.41–0.42) |  | -139.24 |  | <0.001 |
|  |  | 2017–2021 |  | -0.97(-0.99–-0.95) |  | 0.38(0.37–0.38) |  | -123.48 |  | <0.001 |
| Bangladesh |  | 1897–1901 |  | 0.39(0.21–0.57) |  | 1.48(1.23–1.77) |  | 4.24 |  | <0.001 |
|  |  | 1902–1906 |  | 0.40(0.33–0.47) |  | 1.49(1.39–1.60) |  | 11.14 |  | <0.001 |
|  |  | 1907–1911 |  | 0.40(0.35–0.44) |  | 1.49(1.42–1.55) |  | 17.9 |  | <0.001 |
|  |  | 1912–1916 |  | 0.38(0.35–0.42) |  | 1.46(1.42–1.51) |  | 22.25 |  | <0.001 |
|  |  | 1917–1921 |  | 0.37(0.34–0.40) |  | 1.45(1.41–1.49) |  | 24.99 |  | <0.001 |
|  |  | 1922–1926 |  | 0.34(0.31–0.37) |  | 1.40(1.37–1.44) |  | 25.74 |  | <0.001 |
|  |  | 1927–1931 |  | 0.28(0.26–0.30) |  | 1.33(1.30–1.36) |  | 24.29 |  | <0.001 |
|  |  | 1932–1936 |  | 0.23(0.21–0.25) |  | 1.26(1.24–1.29) |  | 22.22 |  | <0.001 |
|  |  | 1937–1941 |  | 0.18(0.16–0.20) |  | 1.20(1.18–1.22) |  | 18.99 |  | <0.001 |
|  |  | 1942–1946 |  | 0.14(0.12–0.16) |  | 1.15(1.13–1.17) |  | 15.73 |  | <0.001 |
|  |  | 1947–1951 |  | 0.10(0.08–0.11) |  | 1.10(1.09–1.12) |  | 12.34 |  | <0.001 |
|  |  | 1952–1956 |  | 0.06(0.05–0.07) |  | 1.06(1.05–1.08) |  | 8.49 |  | <0.001 |
|  |  | 1957–1961 |  | 0.02(0.00–0.03) |  | 1.02(1.00–1.03) |  | 2.68 |  | 0.007 |
|  |  | 1962–1966 |  | -0.02(-0.03–-0.01) |  | 0.98(0.97–0.99) |  | -3.62 |  | <0.001 |
|  |  | 1967–1971 |  | -0.06(-0.07–-0.05) |  | 0.94(0.93–0.95) |  | -12.78 |  | <0.001 |
|  |  | 1972–1976 |  | -0.10(-0.11–-0.10) |  | 0.90(0.89–0.91) |  | -26 |  | <0.001 |
|  |  | 1977–1981 |  | -0.17(-0.18–-0.16) |  | 0.84(0.84–0.85) |  | -49.97 |  | <0.001 |
|  |  | 1982–1986 |  | -0.21(-0.22–-0.21) |  | 0.81(0.80–0.81) |  | -67.3 |  | <0.001 |
|  |  | 1987–1991 |  | -0.23(-0.23–-0.22) |  | 0.80(0.79–0.8) |  | -72.79 |  | <0.001 |
|  |  | 1992–1996 |  | -0.25(-0.26–-0.24) |  | 0.78(0.77–0.78) |  | -74.99 |  | <0.001 |
|  |  | 1997–2001 |  | -0.29(-0.30–-0.29) |  | 0.74(0.74–0.75) |  | -70.28 |  | <0.001 |
|  |  | 2002–2006 |  | -0.34(-0.35–-0.33) |  | 0.71(0.71–0.72) |  | -62.87 |  | <0.001 |
|  |  | 2007–2011 |  | -0.38(-0.39–-0.37) |  | 0.68(0.67–0.69) |  | -51.71 |  | <0.001 |
|  |  | 2012–2016 |  | -0.53(-0.55–-0.51) |  | 0.59(0.58–0.60) |  | -50.16 |  | <0.001 |
|  |  | 2017–2021 |  | -0.70(-0.74–-0.67) |  | 0.50(0.48–0.51) |  | -42.08 |  | <0.001 |
| DRC |  | 1897–1901 |  | 0.28(-0.19–0.76) |  | 1.33(0.82–2.14) |  | 1.16 |  | 0.245 |
|  |  | 1902–1906 |  | 0.34(0.13–0.55) |  | 1.41(1.14–1.74) |  | 3.15 |  | 0.002 |
|  |  | 1907–1911 |  | 0.34(0.22–0.46) |  | 1.40(1.24–1.58) |  | 5.47 |  | <0.001 |
|  |  | 1912–1916 |  | 0.30(0.22–0.38) |  | 1.35(1.25–1.47) |  | 7.35 |  | <0.001 |
|  |  | 1917–1921 |  | 0.26(0.19–0.33) |  | 1.30(1.21–1.39) |  | 7.72 |  | <0.001 |
|  |  | 1922–1926 |  | 0.23(0.17–0.29) |  | 1.26(1.19–1.34) |  | 7.76 |  | <0.001 |
|  |  | 1927–1931 |  | 0.21(0.15–0.26) |  | 1.23(1.16–1.29) |  | 7.57 |  | <0.001 |
|  |  | 1932–1936 |  | 0.17(0.12–0.22) |  | 1.19(1.13–1.25) |  | 7 |  | <0.001 |
|  |  | 1937–1941 |  | 0.14(0.09–0.18) |  | 1.15(1.10–1.20) |  | 6.15 |  | <0.001 |
|  |  | 1942–1946 |  | 0.11(0.06–0.15) |  | 1.11(1.07–1.16) |  | 5.13 |  | <0.001 |
|  |  | 1947–1951 |  | 0.07(0.04–0.11) |  | 1.08(1.04–1.11) |  | 3.94 |  | <0.001 |
|  |  | 1952–1956 |  | 0.04(0.00–0.07) |  | 1.04(1.00–1.07) |  | 2.25 |  | 0.025 |
|  |  | 1957–1961 |  | -0.01(-0.03–0.02) |  | 0.99(0.97–1.02) |  | -0.5 |  | 0.615 |
|  |  | 1962–1966 |  | -0.05(-0.07–-0.03) |  | 0.95(0.93–0.97) |  | -4.1 |  | <0.001 |
|  |  | 1967–1971 |  | -0.08(-0.10–-0.06) |  | 0.92(0.91–0.94) |  | -7.89 |  | <0.001 |
|  |  | 1972–1976 |  | -0.09(-0.11–-0.08) |  | 0.91(0.90–0.93) |  | -11.44 |  | <0.001 |
|  |  | 1977–1981 |  | -0.09(-0.10–-0.07) |  | 0.92(0.91–0.93) |  | -14.22 |  | <0.001 |
|  |  | 1982–1986 |  | -0.07(-0.08–-0.06) |  | 0.94(0.93–0.94) |  | -14.77 |  | <0.001 |
|  |  | 1987–1991 |  | -0.07(-0.08–-0.06) |  | 0.93(0.93–0.94) |  | -19.34 |  | <0.001 |
|  |  | 1992–1996 |  | -0.11(-0.12–-0.10) |  | 0.89(0.89–0.90) |  | -28.22 |  | <0.001 |
|  |  | 1997–2001 |  | -0.16(-0.17–-0.15) |  | 0.85(0.84–0.86) |  | -29.61 |  | <0.001 |
|  |  | 2002–2006 |  | -0.25(-0.26–-0.23) |  | 0.78(0.77–0.79) |  | -32.63 |  | <0.001 |
|  |  | 2007–2011 |  | -0.36(-0.38–-0.34) |  | 0.70(0.69–0.71) |  | -36.53 |  | <0.001 |
|  |  | 2012–2016 |  | -0.50(-0.53–-0.48) |  | 0.60(0.59–0.62) |  | -41.76 |  | <0.001 |
|  |  | 2017–2021 |  | -0.66(-0.69–-0.63) |  | 0.52(0.50–0.53) |  | -44.32 |  | <0.001 |
| DRC:Democratic Republic of the Congo; CI:confidential interval; RR:risk ratio | | | | | | | | | | |

| **Table S5 The forecast results of the incidence of tuberculosis in 8 high-burden countries(/100,000)** | | | | | | | | | | | | | | | | |
| --- | --- | --- | --- | --- | --- | --- | --- | --- | --- | --- | --- | --- | --- | --- | --- | --- |
| Year |  | India |  | Indonesia |  | China |  | The Philippines |  | Pakistan |  | Nigeria |  | Bangladesh |  | DRC |
| 2020 |  | 219.942 (211.692–228.193) |  | 136.004 (127.838–144.169) |  | 42.351 (40.745–43.958) |  | 348.610 (287.106–410.114) |  | 245.621 (237.223–254.018) |  | 189.474 (182.002–196.945) |  | 136.492 (133.079–139.904) |  | 323.915 (317.660–330.171) |
| 2021 |  | 218.770 (206.683–230.858) |  | 131.646 (120.754–142.537) |  | 40.813 (38.763–42.863) |  | 349.493 (255.192–443.793) |  | 237.665 (226.110–249.220) |  | 182.585 (172.349–192.821) |  | 131.863 (127.264–136.462) |  | 318.248 (307.514–328.981) |
| 2022 |  | 217.521 (200.504–234.539) |  | 127.325 (113.033–141.616) |  | 39.319 (36.715–41.923) |  | 351.039 (214.349–487.730) |  | 230.013 (214.538–245.489) |  | 175.897 (162.259–189.535) |  | 127.349 (121.289–133.410) |  | 312.598 (296.493–328.702) |
| 2023 |  | 216.209 (193.487–238.930) |  | 123.063 (104.982–141.145) |  | 37.869 (34.647–41.092) |  | 353.288 (166.586–539.989) |  | 222.658 (202.815–242.500) |  | 169.388 (152.002–186.774) |  | 122.945 (115.265–130.625) |  | 306.941 (284.878–329.004) |
| 2024 |  | 214.840 (185.814–243.866) |  | 118.896 (96.797–140.995) |  | 36.467 (32.590–40.343) |  | 356.300 (112.700–599.900) |  | 215.598 (191.120–240.076) |  | 163.053 (141.740–184.366) |  | 118.655 (109.265–128.044) |  | 301.3 (272.836–329.765) |
| 2025 |  | 213.415 (177.587–249.243) |  | 114.841 (88.598–141.083) |  | 35.114 (30.565–39.662) |  | 360.142 (52.902–667.381) |  | 208.827 (179.551–238.104) |  | 156.908 (131.590–182.226) |  | 114.487 (103.340–125.634) |  | 295.721 (260.496–330.946) |
| 2026 |  | 211.928 (168.883–254.973) |  | 110.871 (80.447–141.294) |  | 33.809 (28.584–39.034) |  | 364.840 (-12.907–742.587) |  | 202.309 (168.151–236.466) |  | 150.959 (121.635–180.283) |  | 110.434 (97.517–123.351) |  | 290.2 (247.945–332.455) |
| 2027 |  | 210.375 (159.758–260.992) |  | 106.973 (72.393–141.553) |  | 32.549 (26.652–38.445) |  | 370.418 (-85.027–825.863) |  | 196.019 (156.954–235.083) |  | 145.189 (111.914–178.464) |  | 106.483 (91.808–121.158) |  | 284.701 (235.221–334.181) |
| 2028 |  | 208.766 (150.269–267.263) |  | 103.156 (64.486–141.827) |  | 31.331 (24.774–37.888) |  | 376.912 (-163.905–917.729) |  | 189.949 (145.987–233.910) |  | 139.581 (102.448–176.714) |  | 102.628 (86.224–119.032) |  | 279.2 (222.360–336.040) |
| 2029 |  | 207.110 (140.458–273.762) |  | 99.443 (56.769–142.116) |  | 30.158 (22.956–37.361) |  | 384.383 (-250.135–1018.901) |  | 184.098 (135.274–232.921) |  | 134.133 (93.260–175.006) |  | 98.873 (80.781–116.964) |  | 273.719 (209.423–338.014) |
| 2030 |  | 205.407 (130.359–280.455) |  | 95.843 (49.272–142.414) |  | 29.031 (21.201–36.860) |  | 392.899 (-344.456–1130.254) |  | 178.463 (124.830–232.095) |  | 128.857 (84.377–173.338) |  | 95.227 (75.496–114.957) |  | 268.303 (196.476–340.130) |
| DRC: Democratic Republic of the Congo; 95% confidence interval in parentheses | | | | | | | | | | | | | | | | |
